# Enhanced Oxidation of Antibiotics by Ferrate Mediated with Natural Organic Matter: Role of Phenolic Moieties

**DOI:** 10.1021/acs.est.3c03165

**Published:** 2023-06-29

**Authors:** Binglin Guo, Junyue Wang, Krishnamoorthy Sathiyan, Xingmao Ma, Eric Lichtfouse, Ching-Hua Huang, Virender K. Sharma

**Affiliations:** † Department of Environmental and Occupational Health, School of Public Health, Texas A&M University, College Station, Texas, 77843, USA; ‡ Department of Civil and Environmental Engineering, Texas A&M University, College Station, Texas 77843, USA; § School of Civil and Environmental Engineering, Georgia Institute of Technology, Atlanta, Georgia 30332, USA; ∥ Aix-Marseille Université, CNRS, IRD, INRAE, College de France, CEREGE, Aix-en-Provence 13100, France

**Keywords:** ferrate, kinetics, electron transfer, phenolic moieties, iron(V) species

## Abstract

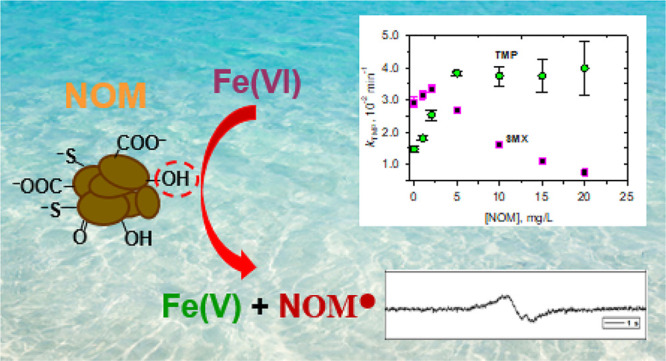

The increasing presence of antibiotics in water sources threatens public health and ecosystems. Various treatments have been previously applied to degrade antibiotics, yet their efficiency is commonly hindered by the presence of natural organic matter (NOM) in water. On the contrary, we show here that nine types of NOM and NOM model compounds improved the removal of trimethoprim and sulfamethoxazole by ferrate(VI) (Fe^VI^O_4_^2–^, Fe(VI)) under mild alkaline conditions. This is probably associated with the presence of phenolic moieties in NOMs, as suggested by first-order kinetics using NOM, phenol, and hydroquinone. Electron paramagnetic resonance reveals that NOM radicals are generated within milliseconds in the Fe(VI)–NOM system via single-electron transfer from NOM to Fe(VI) with the formation of Fe(V). The dominance of the Fe(V) reaction with antibiotics resulted in their enhanced removal despite concurrent reactions between Fe(V) and NOM moieties, the radicals, and water. Kinetic modeling considering Fe(V) explains the enhanced kinetics of antibiotics abatement at low phenol concentrations. Experiments with humic and fulvic acids of lake and river waters show similar results, thus supporting the enhanced abatement of antibiotics in real water situations.

## Abstract 2

Natural organic matter (NOM) at low levels enhances the removal of trimethoprim and sulfamethoxazole by ferrate(VI) through the formation of more reactive Fe(V) species.

## Introduction

The production and consumption of antibiotics continue to increase worldwide because of their increased uses in human health as well as in medical care and disease prevention in livestock and aquaculture.^1^ After their administration, most antibiotics are not completely metabolized, and the undegraded antibiotics are excreted through urine and feces and ultimately enter aquatic environments.^2,3^ The occurrence of antibiotics in the environment has caused public health concerns because elevated antibiotics in the environment induce antibiotic resistant bacteria and genes.^4−6^ In the past two decades, various physical treatment methods, e.g., activated carbon filtration and membrane technology, and chemical oxidation processes, e.g., chlorine, chlorine dioxide, ozonation, Fenton and Fenton-like processes, UV photolysis, and photocatalysis, have been applied to remove antibiotics from water.^1,7−16^ Physical treatments only concentrate antibiotics without their transformation. Thus, advanced oxidation processes (AOPs) may be preferable because they have the potential to break down and even mineralize antibiotics. One potential limit of AOPs is their decreased efficiency in the presence of natural organic matter (NOM).

NOM is a poorly known heterogeneous mixture which derives from the degradation of bacteria, algae, and plant residuals and is ubiquitous in fresh waters.^17^ The concentrations of NOM in fresh water are up to 80 mg/L.^18,19^ NOM contains aliphatic and aromatic moieties, e.g., carbonyl, carboxylic, amines, and phenolics, that may influence the oxidation of micropollutants. Studies on the hydroxyl radical (HO^•^) based AOPs have shown the inhibitory effects of NOM on the abatement of micropollutants because of the high reactivity between HO^•^ and NOM, of 10^8^–10^9^ M^–1^ s^–1^,^20^ and its moieties, e.g., phenol, of ∼10^10^ M^–1^ s^–1^.^21,22^ As a consequence, HO^•^ radicals are often consumed by NOM rather than reacting with the target micropollutants. Iron-based oxidants, predominantly ferrate(VI) (Fe^VI^O_4_^2–^, Fe(VI)), are advantageous in this regard because they are less affected by water constituents.^23^ Fe(VI) has been shown to decrease the concentration of various organic molecules including antibiotics in water.^24−32^ Studies have been conducted to determine the removal efficiency of antibiotics by Fe(VI) in the presence of different cations, anions, and organic constituents.^33−36^ For example, we have studied the influence of inorganic constituents, e.g., chloride, ammonia, and carbonate, and organic components, such as creatine, hippuric acid, and creatinine of urine, in our previous studies.^3,23,37^ We found that ammonia, carbonate, and creatinine enhanced the oxidation of antibiotics by Fe(VI). A few other studies have examined the removal of antibiotics by Fe(VI) in the presence of NOM^31,38,39^ and reported the inhibitory effects of NOM in the removal of micropollutants in water. However, in our study, we observed an enhancing effect of NOM on the Fe(VI) induced abatement of the selected antibiotics trimethoprim and sulfamethoxazole, which are commonly found in contaminated surface water and wastewater. To comprehend this unusual enhancive role of NOM in the oxidation of selected micropollutants by Fe(VI), an in-depth study was carried out in this work.

In the present paper, we hypothesized that NOM or its moieties may generate reactive iron intermediates, iron(V) and/or iron(IV), by reactions with Fe(VI), which would oxidize the target micropollutant more efficiently. For that, we investigated in detail the kinetics of trimethoprim and sulfamethoxazole decrease with Fe(VI) in the presence of NOM model compounds, phenol and hydroquinone, as well as various natural humic and fulvic substances under different reaction conditions.

## Experimental Methods

### Chemicals and Reagents

Trimethoprim, sulfamethoxazole, sulfamonomethoxine (SMMX), sulfachloropyridazine (SCP), sulfadimethoxine (SDM), sulfamethoxypyridazine (SMP), hydroxylamine, phenol, hydroquinone, and disodium phosphate (Na_2_HPO_4_) with high purity (>98%) were obtained from either Sigma-Aldrich (St. Louis, MO, USA) or Fisher-Scientific (Austin, TX, USA). Nine standard NOMs—Nordic Lake I NOM (1R108N), Suwannee River II NOM (2R101N), Suwannee River III FA (3S101F), Suwannee River III HA (3S101H), Suwannee River I NOM (1R101N), Elliott Soil V HA (5S102H), Pahokee Peat II FA (2S103F), Pahokee Peat I HA (1S103H), and Elliott Soil V FA (5S102F)—were purchased from the International Humic Substances Society (IHSS, St. Paul, MN, USA). Other humic acids used were collected from lake water and rivers in Florida. These samples were isolated and purified using standard procedures recommended by IHSS.^40^ High performance liquid chromatography (HPLC) grade methanol and phosphoric acid (85 wt %) were purchased from Merck (Darmstadt, Germany) and Sigma-Aldrich (St. Louis, MO, USA), respectively. A wet chemical synthesis method was used to synthesize potassium ferrate(VI) (K_2_FeO_4_, purity >90%).^1^ The Fe(VI) solution was prepared by dissolving solid K_2_FeO_4_ in 10.0 mM Na_2_HPO_4_ buffer solution. The desired Fe(VI) concentrations were quantified by an UV–visible spectrometer (Evolution 60s, Thermo Scientific Co., USA) at a wavelength of 510 nm with a molar absorption coefficient of ε_510 nm_ = 1150 M^–1^ cm^–1^.^41^

### Measurement of Optical Properties of NOMs

Different types of NOMs were dissolved in DI water to make 200.0 mg/L stock solutions in 10.0 mM phosphate buffer (Na_2_HPO_4_). The dissolution took 24.0 h, and the solutions were filtered through prewashed 0.45 μm poly(ether sulfone) syringe filters (Millipore Sigma, USA). The stock solutions were diluted to 10.0 mg/L in 10.0 mM phosphate buffer, and the pH was adjusted to 9.00 ± 0.02. The concentrations of NOMs in our study are reported in total mass. Total organic carbon is 50% of the total mass in the samples. Absorbance spectra of NOM solutions were obtained using a 1 cm quartz cuvette through full-wavelength (800–200 nm) scans in an UV–visible spectrometer (Evolution 60s, Thermo Scientific Co., USA). Prior to the measurements, the baseline was corrected by scanning a 10.0 mM Na_2_HPO_4_ solution. The E2/E3 values were calculated by dividing the absorbance at 250 nm by the absorbance at 365 nm.^42^ The phenolic content in different NOM samples was determined by the Fourier transform infrared technique (FT-IR). Briefly, 1.0 mg of NOM sample was mixed with 100 mg of potassium bromide and ground to a fine powder with a mortar and pestle, pelletized, and subjected to FT-IR analysis. The FT-IR spectrometer was equipped with an attenuated total reflectance (ATR) module. Transmittance (%) data was measured with the spectral range from 650 to 4000 cm^–1^ with a scan number of 64 and a resolution of 16 cm^–1^. The samples were prepared in triplicate. The phenolic contents in the NOM samples were determined by integrating the area under the characteristic peak at 1457 cm^–1^.^43^ The phenolic contents of three standard NOM samples were known and were used to construct the calibration curve.

### Abatement of Antibiotics in the Presence of NOM

Batch experiments were carried out in 60.0 mL glass beakers. Trimethoprim, sulfamethoxazole, and other antibiotic solutions at a concentration of 10.0 μM were prepared by adding the corresponding solids in 10.0 mM Na_2_HPO_4_ buffer. For investigating the role of NOM in the decay of trimethoprim and sulfamethoxazole at low concentrations, the stock solutions were diluted to 2.0 μM. The stock solutions of 200.0 mg/L NOM were prepared in 10.0 mM Na_2_HPO_4_ buffer, and the pH was adjusted to a desired level for conducting the experiments at pH 7.0, 8.0, and 9.0, respectively. Before mixing with 200.0 μM Fe(VI), a certain amount of NOM stock was first mixed with either trimethoprim or sulfamethoxazole solutions. All reactions were performed at a constant temperature of 23.0 ± 0.2 °C. In the kinetics study, an aliquot of 1.0 mL of reactant solution was withdrawn periodically. The remaining amount of Fe(VI) in the reactant mixture was quenched by a 10.0 μL NH_2_OH solution (1.0 M, [NH_2_OH]:[Fe(VI)] ≥ 10.0) in the 1.5 mL high performance liquid chromatography (HPLC) vials (Ultimate 3000, ThermoFisher Scientific). The concentrations of trimethoprim or sulfamethoxazole in samples were determined using HPLC methods as described below.

### Abatement of Antibiotics in the Presence of Phenol and Hydroquinone

In this study, the stock solutions in 2.0 mM phenol or 2.0 mM hydroquinone were prepared by dissolving the corresponding solids in 10.0 mM Na_2_HPO_4_ buffer. The mixing procedures and pH adjustment as well as the analysis of antibiotics were the same as described above.

### Analysis of Antibiotics

The concentrations of trimethoprim and sulfamethoxazole were analyzed using an HPLC with a RESTEK Ultra C18 analytical column (4.6 mm × 250 mm, particle size 5 μm) at 30 °C. The mobile phases were (A) 0.5 wt % phosphoric acid–water solution and (B) 100% methanol. More details of the conditions of HPLC methods are given in Table S1.^44^

### Determining Rate Constants of Phenol with Fe(VI)

A series of phenol solutions in the range 20–80 mM were prepared in 10.0 mM Na_2_HPO_4_ buffer solution. The concentration of Fe(VI) was kept at 200.0 μM at pH 9.0 buffered in 10.0 mM Na_2_HPO_4_ solution. For the reaction at pH 8.0, the phenol solution was adjusted to pH 7.6 before mixing. A stopped-flow spectrophotometer (SX.18 MV, Applied Photophysics, U.K.) was applied to mix various phenol solutions with Fe(VI), and the absorbance at 510 nm was recorded for determining the Fe(VI) decay. Since [phenol]_0_ ≫ [Fe(VI)]_0_, the pseudo-first-order rate constants (*k*_obs_) at different [phenol]_0_’s were fitted to exponential decay kinetics and observed *k*_obs_ was plotted versus [phenol]_0_; the slope of the plot gave the second-order rate constant for the reaction between Fe(VI) and phenol.

### Electron Paramagnetic Resonance (EPR) Measurements

Samples of EPR were prepared by mixing solutions from each syringe (equal volumes). The mixed solution was quenched by freezing at the selected time following mixing. In the case of samples frozen in less than 1 s after mixing, quenching was achieved by spraying the mixed solution directly into liquid solvent (−150 °C) using a System 1000 Chemical/Freeze Quench Apparatus (Update Instruments, Inc.). The length of the aging loop determined the reaction time. A modified flow–pause–flow freeze–quench procedure was used for preparing samples for reaction between 1 and 20 s. All samples were stored in liquid N_2_ prior to the collection of EPR spectra. A low temperature EPR spectrum was obtained on a Bruker EMX spectrometer, equipped with an Oxford Instrument liquid helium cryostat. The spectra were collected at 9.6 GHz frequency.

### Kinetic Modeling

The concentration decrease of trimethoprim and sulfamethoxazole in the Fe(VI)–phenol system was modeled with reactions R1–R14 (Table 1) using the Kintecus program 4.55.31. Briefly, the reaction kinetics between Fe(VI) and sulfamethoxazole/trimethoprim, without phenol, were first simulated by the FIT:2:3:FITDATA.TXT command on Kintecus. Then, the reaction kinetics between Fe(V) and trimethoprim/sulfamethoxazole were simulated by their decrease in the presence of phenol (0.1–5.0 μM). The Fe(VI)–NOM system was not simulated due to the lack of rate constants related to NOM. The goodness of fit between simulation and experimental data was quantified by calculating the normalized root-mean-square deviation (RMSD).

## Results

### Decrease of Antibiotic Levels in the Presence of NOM

**Figure 1:**
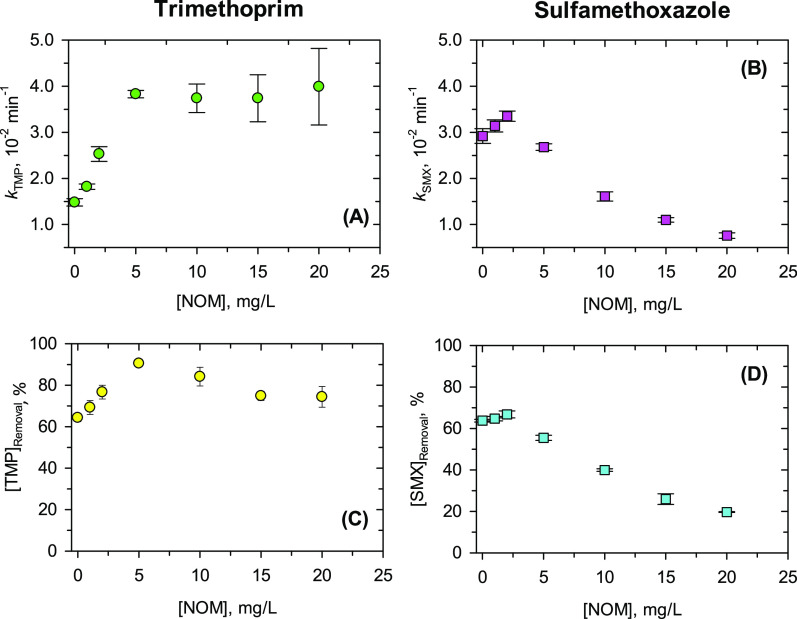
Effects of different NOM concentrations on decrease in concentrations of trimethoprim (TMP) and sulfamethoxazole (SMX). (A) First-order constant of abatement of trimethoprim (*k*_Trimethoprim_, min^–1^), (B) first-order constant of sulfamethoxazole removal (*k*_Sulfamethoxazole_, min^–1^), (C) percent removal of trimethoprim at 60 min, and (D) percent removal of sulfamethoxazole at 30.0 min. Experimental conditions: [trimethoprim]_0_ = [sulfamethoxazole]_0_ = 5.0 μM; [Fe(VI)]_0_ = 100.0 μM; pH 9.0 buffered by 10.0 mM Na_2_HPO_4_; Suwannee River NOM was used here.

In this set of experiments, the concentration of trimethoprim (TMP) or sulfamethoxazole (SMX) by Fe(VI) was followed as a function of time in the presence of 0.0–20.0 mg/L NOM at pH 9.0 (Figure 1). The results show an enhanced abatement of both antibiotics with an increasing amount of Suwannee River natural organic matter (NOM) at relatively low concentrations, followed by either no further enhancement, i.e., for trimethoprim, or inhibition, i.e., for sulfamethoxazole, of the oxidation by Fe(VI) at higher concentrations (Figure S1). The concentration drop is satisfactorily fitted by first-order kinetics up to 10.0 mg/L NOM, with *r*^2^ values of 0.98–0.99 (Tables S2 and S5). The maximum first-order rate constant for the decrease of trimethoprim concentration (*k*_TMP,NOM_) of (3.83 ± 0.08) × 10^–2^ min^–1^ was observed at 5.0 mg/L NOM, and for sulfamethoxazole, the maximum *k*_SMX,NOM_ of (3.35 ± 0.11) × 10^–2^ min^–1^ was achieved at 2.0 mg/L NOM. At NOM concentrations of 10.0–20.0 mg/L, the decreasing kinetics negatively deviated from the first order, with *r*^2^ values of 0.88–0.97, possibly due to the relatively low concentration of Fe(VI) in the mixtures.

The dependence of the oxidation rate constants of trimethoprim and sulfamethoxazole on the levels of NOM is shown clearly in Figure 1A,B. In the oxidation of trimethoprim, the enhancing effect of NOM was observed at all studied levels with a somewhat linear increase up to 5.0 mg/L NOM. In the case of sulfamethoxazole, the enhancement was only up to 2.0 mg/L NOM; then inhibitory effects were seen in the range 5.0–20.0 mg/L. The kinetics of trimethoprim and sulfamethoxazole influenced their removal percentages, as shown in Figure 1C,D at 30 min reaction time. The maximum removal of trimethoprim reached ∼91% at 5.0 mg/L NOM. Without NOM, the removal of trimethoprim was 64% (Table S2). The removal percentage of sulfamethoxazole at different levels of NOM was mostly inhibitory (Figure 1D and Table S5). This suggests that the type of antibiotics may be of importance in responding to the effects of NOM on their decreasing kinetics and removals by Fe(VI).

Next, the impacts of NOM types on the removal efficiency of trimethoprim and sulfamethoxazole were investigated. Nine different NOMs at 10.0 mg/L were tested, in which the reactants were allowed to mix for 60 min and then the concentrations of the antibiotics were determined. The results are given in Table S6. Significantly, the NOM-enhanced removal of trimethoprim and sulfamethoxazole by Fe(VI) varied with the nature of organic matter, ranging 50–64% and 9–36% for trimethoprim and sulfamethoxazole, respectively (Table S6). The cause of such variation in removal was explored by correlating removals with the physicochemical properties of organic matter, which are summarized in Table S7. Most of the properties, including the ash percentage, H/C and O/C ratios, and contents of carbonyl, aromatic, acetal, heteroaliphatic, and aliphatic groups, showed poor relationships with the removal of trimethoprim and sulfamethoxazole (Figures S2 and S3).

**Figure 2:**
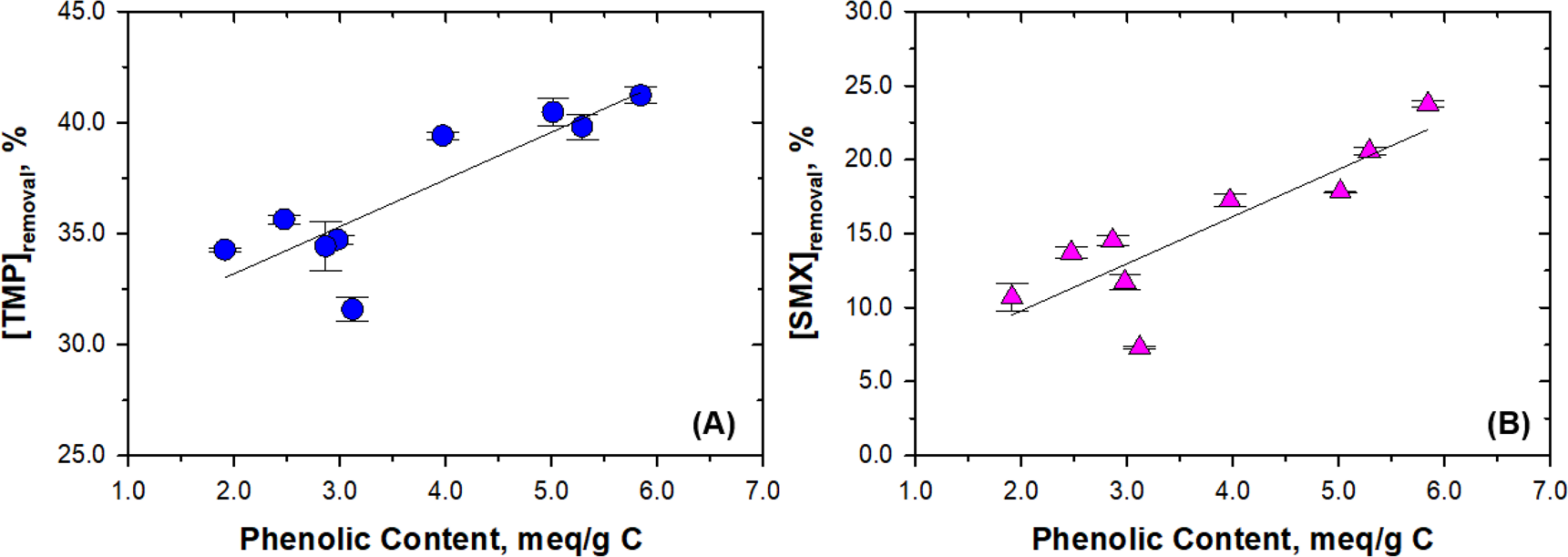
Relationship between phenolic contents of different NOMs and (A) removal percentage of trimethoprim (TMP) at 30.0 min and (B) removal percentage of sulfamethoxazole (SMX) at 15 min. Experimental conditions: [trimethoprim]_0_ = [sulfamethoxazole]_0_ = 5.0 μM; [NOM] = 10.0 mg/L; [Fe(VI)]_0_ = 100.0 μM; pH 9.0 buffered by 10.0 mM Na_2_HPO_4_; reaction time = 60.0 min.

The positive influences of carboxyl groups like acetate and peracetate present in pure water on the abatement of pharmaceuticals by Fe(VI) have been reported. However, in our current study, the removal of trimethoprim and sulfamethoxazole was unaffected by the carboxyl content (Figure S4A,B). Similarly, no significant relationship is seen in Figures S2E and S3E.^45,46^ A similar observation is seen in the correlation of removal efficiency with the ratio of E2/E3 (Figure S4C,D). E2/E3 gives information on molecular weight fractions of organic matter;^42,47^ hence, the removal efficiency of TMP and SMX was not related to the molecular weight fraction of the natural organic matter. In contrast, the phenolic content of the organic matter showed a positive trend (*r*^2^ = 0.7304 and 0.7324 for trimethoprim and sulfamethoxazole, respectively) with the removal efficiency of both trimethoprim and sulfamethoxazole (Figure 2).

Overall, the decrease in antibiotics by Fe(VI) was enhanced by NOM at low concentrations and then was inhibited at a high NOM level. The phenolic content of NOM is most likely involved in the enhancement of the antibiotic decrease by NOM. The role of phenolic content of organic matter was thus further investigated by carrying out independent studies on the decrease of antibiotics in the presence of phenol and hydroquinone, and the results are described in the next section.

### Decrease of Antibiotic Concentrations in the Presence of Phenol and Hydroquinone

**Figure 3:**
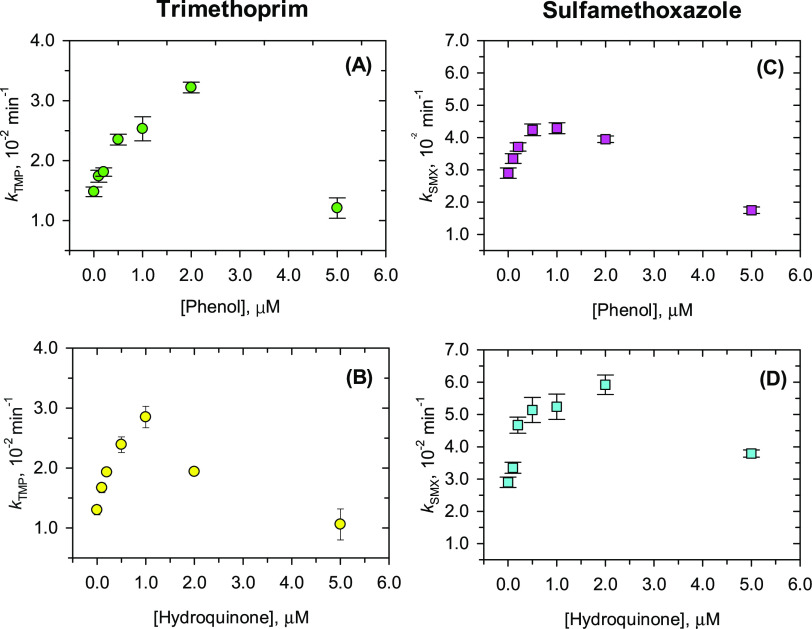
Effects of phenolic model compounds of NOM on first-order rate constant of the abatement of antibiotic concentrations by Fe(VI), i.e., the decrease in concentrations of trimethoprim (TMP) as affected by different concentrations of (A) phenol and (B) hydroquinone and the decrease of sulfamethoxazole (SMX) in the presence of different concentrations of (C) phenol and (D) hydroquinone. Experimental conditions: [trimethoprim]_0_ = [sulfamethoxazole]_0_ = 5.0 μM; [Fe(VI)]_0_ = 100.0 μM; pH 9.0 buffered by 10.0 mM Na_2_HPO_4_; reaction time = 60.0 min for trimethoprim and 30.0 min for sulfamethoxazole.

Decreases of trimethoprim and sulfamethoxazole concentrations at different concentrations of phenol and hydroquinone were monitored over time at pH 9.0 (Figure S5). The decay of trimethoprim and sulfamethoxazole with time fitted nicely to the first-order kinetics at low concentrations of phenol/hydroquinone (Tables S8–S11). The variations of the first-order rate constants with concentrations of phenol and hydroquinone are presented in Figure 3. The patterns of *k*_TMP_ and *k*_SMX_ variation with the concentrations of phenol and hydroquinone are similar to the trends seen in the presence of NOM (Figure 1). The enhancement was also observed at lower concentrations of phenol and hydroquinone, but further increase of phenol and hydroquinone levels beyond an optimal concentration resulted in a decrease in the pseudo-first-order rate constants. The values of the rate constants were of the same order of magnitude for both compounds at the optimal concentrations of phenol and hydroquinone, i.e., *k*_TMP,Phenol_ of (3.22 ± 0.09) × 10^–2^ min^–1^ at 2.0 μM phenol, *k*_TMP,Hydroquinone_ of (2.85 ± 0.18) × 10^–2^ min^–1^ at 1.0 μM hydroquinone, *k*_SMX,Phenol_ of (4.29 ± 0.17) × 10^–2^ min^–1^ at 1.0 μM phenol, and *k*_SMX,Hydroquinone_ of (5.92 ± 0.30) × 10^–2^ min^–1^ at 2.0 μM hydroquinone.

Results shown in Figure 2 suggest the dominating role of phenolic moieties of the organic matter in affecting the oxidation of antibiotics by Fe(VI). However, the decreasing trend of the rate constants for oxidizing trimethoprim in the presence of phenol and hydroquinone was seen at high phenol concentrations, which was not the case in oxidizing this antibiotic in the presence of NOM (Figure 3A,C versus Figure 1A). Also, the decrease in removal efficiency of sulfamethoxazole in the presence of phenol and hydroquinone was not as sharp as those in the presence of NOM (Figure 3B,D versus Figure 1B). This indicates that other factors besides phenolic moieties of NOM, such as competing reactions, may also contribute to the decrease in concentrations of trimethoprim and sulfamethoxazole in the presence of organic matter. This will be discussed further in Discussion.

The effect of pH on the enhanced oxidation kinetics and removal of trimethoprim was also investigated by lowering the pH from 9.0 to 8.0 and 7.0. The calculated first-order rate constants and the removal of trimethoprim at various concentrations of NOM and phenols at pH 8.0 and 7.0 are given in Tables S3 and S4. The decrease in concentrations of trimethoprim at pH 8.0 and 7.0 were faster than that at pH 9.0 (*k*_TMP_ ∼ 10^–1^ min^–1^ at pH 8.0 and 7.0 versus *k*_TMP_ ∼ 10^–2^ min^–1^ at pH 9.0). This is in agreement with earlier reports that lowering pH usually increases the reaction rate of Fe(VI) with pollutants.^23,48^ Furthermore, independent kinetic measurements on the oxidation of trimethoprim and sulfonamides have also shown increased removal with a decrease in pH from alkaline to acidic medium.^49,50^

**Figure 4:**
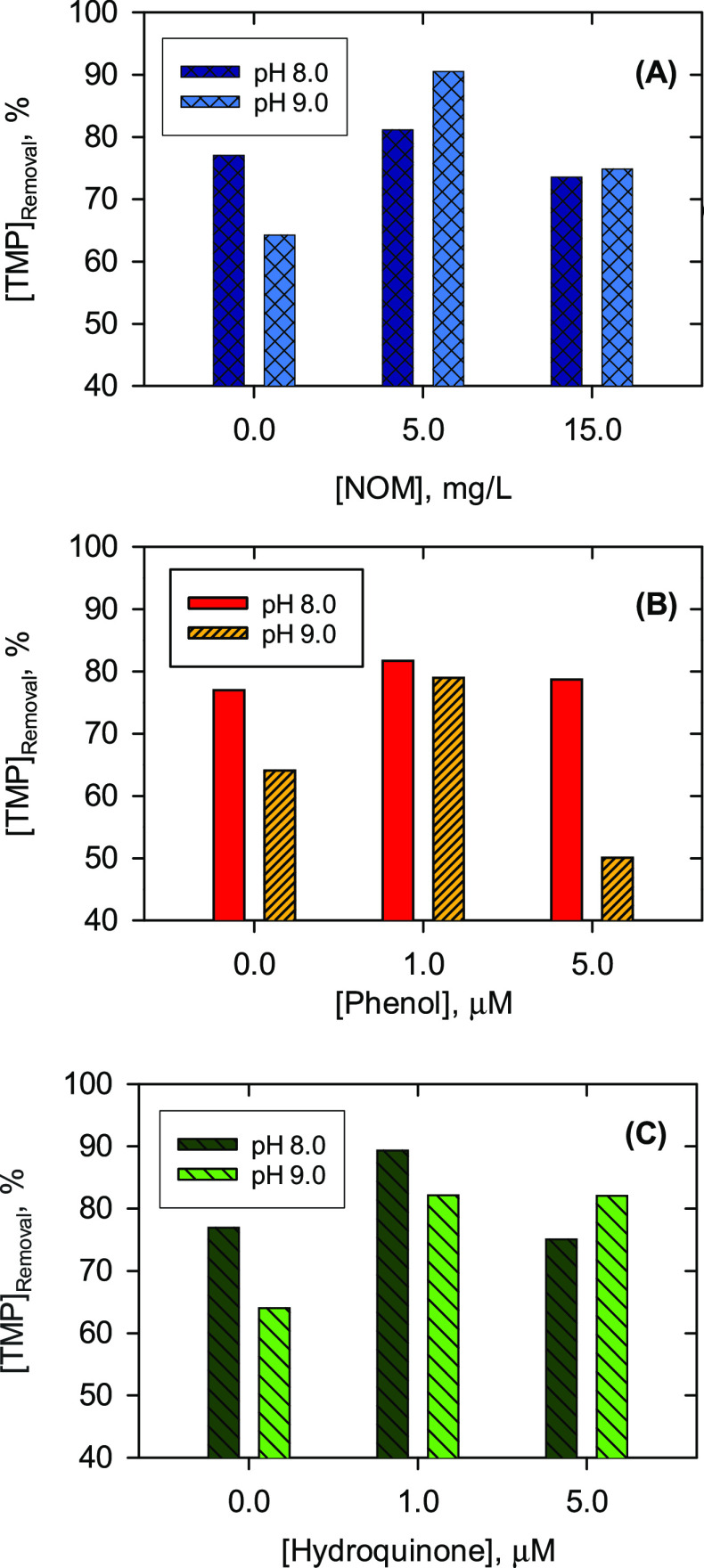
Effects of pH on percent removal of trimethoprim (TMP) by Fe(VI) in the presence of (A) NOM, (B) phenol, and (C) hydroquinone. Experimental conditions: [trimethoprim]_0_ = 5.0 μM; [Fe(VI)]_0_ = 100.0 μM; solution was buffered by 10.0 mM Na_2_HPO_4_; reaction time = 60.0 min.

The dependence of the rate constants of trimethoprim decay on the concentrations of NOM at pH 7.0 and 8.0 and of phenol and hydroquinone at pH 8.0 is presented in Figures S8 and S9. The *k*_TMP_ did not show much variation with concentrations of NOM at pH 7.0 and 8.0 (Figure S8) and phenol at pH 8.0 (Figure S9A), which is different from the results at pH 9.0 (see Figures 1 and 3). In using hydroquinone, a similar trend in the oxidation of trimethoprim by Fe(VI) at pH 9.0 (Figure 3B and Figure S9B) was observed. Results of pH dependence suggest that various competing reactions are involved in trimethoprim removal by Fe(VI) in the presence of NOM, and the influence of pH on the rate constants of these involved reactions in the system greatly differs (see Discussion). The effect of pH on the removal of trimethoprim at selective concentrations of organic matter and phenols can be seen in Figure 4. Without organic matter, the removal of trimethoprim was higher at pH 8.0 than at pH 9.0, as expected (Figure 4A). However, the removal was not significantly affected by pH in the presence of 1.0 and 5.0 mg/L NOM. This trend was generally true for the removal of trimethoprim by Fe(VI) in the presence of phenol and hydroquinone as well (Figure 4B,C). The exception was for phenol at 5.0 mg/L, which resulted in a decrease in trimethoprim removal from 79% at pH 8.0 to 50% at pH 9.0 (Figure 4B). Compared with the above-mentioned similar trimethoprim removal efficiency at different pHs in the presence of NOM, the result again indicated that additional parameters, such as types and concentrations of functional groups, in addition to phenolic moieties, could affect the overall removal of the antibiotics by Fe(VI) in the presence of NOM.

## Discussion

The enhanced decrease of antibiotics by Fe(VI) in the presence of NOM may be understood by considering the possible reactions in the Fe(VI)–trimethoprim/sulfamethoxazole–NOM mixture. According to the correlation between phenolic contents and the enhancing effect of NOM, an attempt was made by using a simple molecule, i.e., phenol, as a representative model of NOM, based on the results of Figure 3. Table 1 gives the postulated reactions R1–R14 with the reported rate constants at pH 9.0 that could occur in the Fe(VI)–trimethoprim/sulfamethoxazole–phenol (C_6_H_5_OH) solution.^31,51−55^ As shown in Table 1, different oxidants, i.e., Fe(VI) and Fe(V), may yield different oxidation products of trimethoprim and are presented as OP_T_′ and OP_T_″, respectively (reactions R1 and R4). It is noteworthy that Fe(IV)/Fe(V) may be generated by single- and/or double-electron transfer between TMP and ferrate(VI), which are difficult to distinguish and kinetically simulate. However, as phenol reacts with ferrate(VI) much faster than TMP, the Fe(IV)/Fe(V) generated by TMP should be negligible and reaction R1 is the simplified reaction between Fe(VI) and TMP. Similarly, the involved reactions of Fe(VI) and Fe(V) with phenol and phenoxide ions are presented as OP_1_, OP_2_, OP_3_, and OP_4_. In the absence of phenol, trimethoprim was oxidized by Fe(VI) (reaction R1). The reaction between Fe(VI) and trimethoprim (R1) has been well studied,^48^ and its rate constant at pH 9.0 was simulated to be 2.7 M^–1^ s^–1^ in this study. In presence of phenol, additional reactions may happen (R2 and R3). Note that the self-decay of Fe(VI) could be neglected at pH 9.0, compared to Fe(VI) reduction by NOM, phenol, or hydroquinone. Further, the generation of Fe(IV)/Fe(V) by trimethoprim or sulfamethoxazole reduction of Fe(VI), if any, could also be neglected in the presence of other activators (e.g., phenol). Thus, these reactions are not included in the kinetic model (Table 1).

**Table 1:** Kinetic Model for the Fe(VI)–Phenol System at pH 9.0

no.	reaction	*k* (M^–1^ s^–1^)	ref
R1	Fe(VI) + trimethoprim → Fe(III) + OP_T_′	2.7	simulated
R2	Fe(VI) + C_6_H_5_OH → Fe(V) + C_6_H_5_O^•^	1.1 × 10^2^	(51)
R3	Fe(VI) + C_6_H_5_O^•^ → Fe(V) + OP_1_	1.0 × 10^9^	(51−54)
R4	Fe(V) + trimethoprim → Fe(III) + OP_T_″	3.8 × 10^6^	simulated
R5	Fe(V) + H_2_O → Fe(III) + H_2_O_2_	5.0	(31)
R6	2 Fe(V) → 2 Fe(III) + 2 H_2_O_2_	1.5 × 10^7^	(31)
R7	Fe(VI) + H_2_O_2_ → Fe(IV) + O_2_	negligible	(31)
R8	Fe(V) + H_2_O_2_ → Fe(III) + O_2_	4.0 × 10^5^	(31)
R9	Fe(V) + C_6_H_5_OH → Fe(III) + OP_2_	1.0 × 10^7^	(51)
R10	Fe(V) + C_6_H_5_O^•^ → Fe(IV) + OP_3_	not included	(51)
R11	Fe(V) + 2 C_6_H_5_O^•^ → Fe(III) + OP_4_	not included	(51)
R12	C_6_H_5_O^•^ + C_6_H_5_O^•^ → HO–(C_6_H_4_)–OH	2.2 × 10^9^	(51)
R13	Fe(VI) + sulfamethoxazole → Fe(III) + OP_S_′	4.6	simulated
R14	Fe(V) + sulfamethoxazole → Fe(III) + OP_S_″	2.0 × 10^6^	simulated

The reaction of Fe(VI) with phenol, i.e., reaction R2 in Table 1, has been studied by many researchers.^51−54,56,57^ The value of *k*_2_ has been reported to be 1.1 × 10^2^ M^–1^ s^–1^,^51^ which is independent of pH in the range from 5.0 to 9.0. We have also determined the values of *k*_2_ at pH 8.0 and 9.0 and found similar values (Figure S10). Significantly, an one-electron-transfer step of reaction R2 was proposed to form Fe(V) and phenoxide radical (C_6_H_5_O^•^).^51^ The formation of radicals in reaction R2 was confirmed by EPR measurements.^51^ The radicals may further react with Fe(VI) to give another Fe(V) (R3). Generally, Fe(VI) reacts with the aromatic radicals at ∼10^9^ M^–1^ s^–1^.^58^ Fe(V) usually reacts 3–5 orders of magnitude faster with organic compounds than does Fe(VI).^55,59^ Importantly, the formed Fe(V) in reactions R2 and R3 due to phenol in the reaction mixture would react with trimethoprim to cause enhanced decontamination (R4). The generated Fe(V) may also react simultaneously with water by first- and second-order kinetics and yield hydrogen peroxide (H_2_O_2_) (R5 and R6). The reactions of Fe(VI) and Fe(V) with H_2_O_2_ release oxygen (R7 and R8).^60^ As a result, we simulated the enhancement of trimethoprim removal with *k*_4_ at 3.8 × 10^6^ M^–1^ s^–1^ (Figure S11), and the goodness of fit is shown in Table S13. The model captured the trend of trimethoprim removal with up to 1.0 μM phenol but was unable to simulate trimethoprim removal at a high phenol concentration, above 5.0 μM, suggesting that the competitive consumption of Fe(V) by extra phenol and its transformation products was underestimated by the model (will be discussed later).

The values of *k*_TMP,Phenol_ at higher concentrations of phenol decreased, which indicates that the consumption of produced Fe(V) by excessive phenol and/or its oxidation products. The reaction between Fe(V) and phenol (R9, Table 1) has been investigated by a premix pulse radiolysis technique, and a two-electron-transfer step was suggested as no characteristic spectrum of Fe(IV) was observed.^60^ Another possibility of the consumption of Fe(V) is its reaction with phenoxide radical (R10 and R11, Table 1), which are proceeded by two-electron-transfer steps based on the experimentally determined oxidized products of phenol.^52^ However, considering that Fe(VI) has a high reactivity with phenol radical, and the concentration of Fe(VI) is much higher than that of Fe(V), phenol radicals should be mainly consumed by Fe(VI) and their reaction with Fe(V) could be neglected. There is also a possibility that the phenoxide radical decays itself by bimolecular rate constants (R12, Table 1).

Overall, reactions R9–R11 are undesirable in the Fe(VI)–trimethoprim–phenol system for the decrease in level of trimethoprim. Therefore, a phenol dosage at 5.0 μM or higher inhibited trimethoprim removal. The kinetic model could not simulate the inhibitory effect of 5.0 μM phenol, indicating that Fe(V) consumption by phenol and its oxidation products was still underestimated (Figure S11). We found the products from reaction R12 were too little to affect Fe(V) concentration in the model; however, the other identified product, 1,4-benzoquinone,^52^ may consume Fe(V) and inhibit trimethoprim removal. Nonetheless, the reaction pathways and rates of Fe(VI)/Fe(V) with benzoquinone are currently unavailable. Thus, these reactions were not included in the kinetic model. Similar reactions as shown in reactions R10–R12 would happen in the presence of hydroquinone; hence, a similar pattern of the decrease of trimethoprim by the Fe(VI)–trimethoprim–hydroquinone system was observed (see Figure 3B).

In the decrease in concentration of sulfamethoxazole in the presence of phenol or hydroquinone, reactions R1 and R4 would be replaced by reactions R13 and R14, while other reactions remained the same (Table 1). Here oxidized products of sulfamethoxazole reactions with Fe(VI) and Fe(V) are assigned as OP_S_′ and OP_S_″, respectively. In the absence of phenol, only reaction R13 would occur and the decrease of sulfamethoxazole is faster than that of trimethoprim,^61,62^ which could be noticed in higher *k*_SMX_ than *k*_TMP_ (Figure 3, part C versus part A). The variations of *k*_SMX,Phneol_ and *k*_SMX,Hydroquinone_ with the concentrations of phenol and hydroquinone were similar to the observed decrease of trimethoprim. Similarly, our model could simulate the enhancement by phenol at ≤2.0 μM phenol concentration but not the inhibition with ≥5.0 μM phenol (Figure S11), due to the knowledge gap of Fe(V) consumption by the oxidation products of phenol (e.g., 1,4-benzoquinone).

The results in Figure 4 may be understood by considering the variations of rate constants of reactions R1–R14 with pH. The concentration of generated Fe(V) from reaction R2 and its competitive reaction rate constants with trimethoprim (R4) and phenol (R9) would generally determine the overall effect of removal of trimethoprim (or sulfamethoxazole) by Fe(VI) in the presence of phenol (i.e., enhancement reaction R4 versus inhibitory reaction R9). Because the rate constant for reaction R9 does not vary with pH in the range 5.0–11.0,^51^ the observed effect of pH removal of trimethoprim in the presence of NOM, phenol, and hydroquinone may thus be related to the variation of rates of reaction R4. The rate constants of the reaction of Fe(VI) with nitrogen-containing organic compounds usually increased with a decrease in pH,^35,50^ and this analogy may explain the results of higher enhanced effects of removal of trimethoprim at pH 8.0 than at pH 9.0 without phenol. However, the rate constants of reaction R4 at different pHs are needed to fully describe the results of Figure 4.

In the presence of NOM, the formation of Fe(V) and the radical in the initial step (eq 1) influenced the observed effects of NOM on the oxidation of antibiotics.
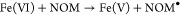
1

**Figure 5:**
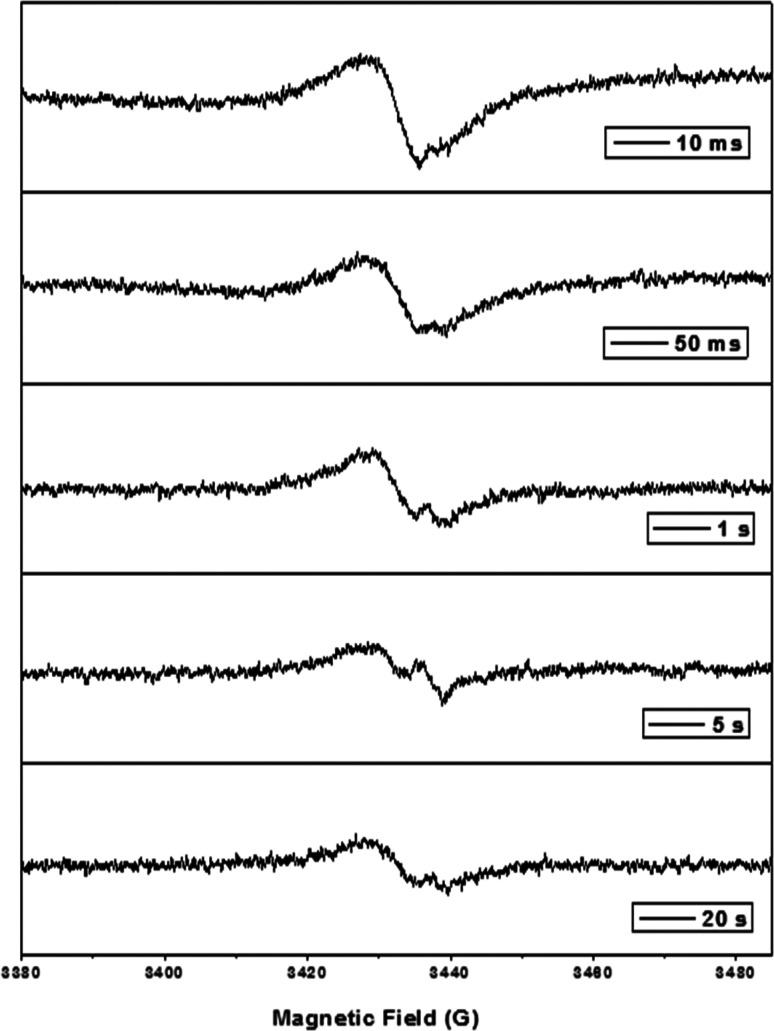
Formation of radical(s) in the reaction of Fe(VI) with Suwannee River humic acid (SRHA) mixture at varying reaction time (pH 8.0).

The experimental evidence of eq 1 was sought by performing EPR measurements of the mixture of Fe(VI) with humic acid (Figure 5). The radical formed in a millisecond time scale and was subsequently converted to another radical. Figure 5 shows the formation of this radical in a second time scale. The type of the radical generated in the Fe(VI)–NOM mixture may depend on the ratio of the concentration of Fe(VI) to NOM. Significantly, Fe(V) may not be the only oxidative species; the radical (NOM^•^) may acquire oxidative character to participate in oxidizing the antibiotics. The reaction of Fe(VI) with NOM^•^ to generate Fe(V) is crucial for the enhanced oxidation of antibiotics. In particular, phenolic moieties in NOM play an important role in generating Fe(V) and contribute to the oxidation of antibiotics, especially at low levels of NOM (see Figure 1A,B). However, at a higher level of NOM, effects of enhancement and inhibition on the decrease of trimethoprim and sulfamethoxazole were observed, respectively, suggesting complicated roles of NOM in influencing Fe(VI) to oxidize antibiotics in water. In Table 1, the possibility of the reaction of antibiotic radical, generated from the reaction between Fe(VI) and the targeted antibiotic, with the moieties of NOM was ruled out because of the preference of Fe(VI) for such radicals, which have rate constants of 10^8^–10^9^ M^–1^ s^–1^.^58,59^

## Environmental Significance

Results demonstrate that NOM levels influenced the abatement of antibiotics by Fe(VI). The enhancive effect was also tested by lowering the concentrations of trimethoprim and sulfamethoxazole from 5.0 to 1.0 μM (Figure S13). The enhancement in trimethoprim and sulfamethoxazole (1.0 μM) removal was still seen at a low level of NOM (1.0 mg/L), suggesting the concentration of NOM and amount of Fe(VI) likely derived the enhancement of the removal of antibiotics. Furthermore, NOM could enhance antibiotic oxidation at environmentally relevant concentrations (e.g., ∼1 μM). An enhancive effect of organic matter in a broad range of concentrations was observed for trimethoprim removal. However, only a narrow range of the levels of organic matter could result in similar enhancement for the removal of sulfamethoxazole.

The finding of our study was further tested using humic acids (HAs) and fulvic acid (FA) from lakes and rivers of Florida. Removals of trimethoprim and sulfamethoxazole were investigated in the presence of HA and FA at the level of 10.0 mg/L. The difference (Δ) of removal without and with HA and FA is shown in Figure S12. Removal of trimethoprim was enhanced, while the removal of sulfamethoxazole was inhibited in the presence of organic matter. The results are consistent with the removal in the presence of NOM (Figure 1C,D). The enhancing role of low concentration NOM on the removal of trimethoprim was observed at different pHs. The extent of enhancement varies at different pHs, which alludes to the important role of NOM in the treatment of antibiotics at all pHs. However, the complexity of the effects at different pHs is involved due to the pH dependence of reactions involved in the oxidative system containing Fe(VI)–NOM–antibiotics.

The effects of NOM on the decrease of other sulfonamides by Fe(VI) were also tested (Figure S14). Significant enhancement of the removal of SMMX and SCP was observed at 15 min in the presence of 1.0 mg/L NOM. However, the removals of SDM and SMP were not significantly affected by the presence of NOM. This implies again that the structure of antibiotics is an important consideration in their abatement in water bodies by Fe(VI). The generation and amount of the highly reactive species Fe(V) are imperative in contributing to the decrease of antibiotics. The moieties (like phenolic groups) of the organic matter produced Fe(V) from Fe(VI). When Fe(V) could react with the target antibiotics, increased oxidation rates were found. However, other competitive reactions (i.e., Fe(V) with phenol (or organic matter), phenoxide radical (or organic matter radical), and water) could result in inhibitory effects of NOMs on the abatement of antibiotics in natural water bodies. Our study highlighted the complexity of the removal of antibiotics in natural water but suggested that mechanistic understanding of the complex reactions involved in the removal of different antibiotics in natural water bodies could lead to better control of the reaction conditions and more efficient removal of antibiotics by Fe(VI). Finally, the oxidized products of the antibiotics by Fe(VI) and their antibacterial activities have been investigated, which suggests a decrease in activities after Fe(VI) treatment.^25,49,62−64^

## Supporting Information Available

Kinetic decays of TMP and SMX in NOM, phenol, and hydroquinone; relationships of removals of TMP and SMX with physicochemical properties of nine standard NOMs; first-order rate constants for decrease in concentrations of TMP as a function of concentrations of NOM, phenol, and hydroquinone; second-order rate constant of reaction of Fe(VI) with phenol at pH 8.0 and 9.0; kinetic modeling results of decrease of TMP and SMX in the presence of different phenol concentrations; removal of TMP and SMX with and without humic and fulvic acids; effect of 1.0 mg/L NOM on the abatement of concentration of TMP and SMX at environmental related level; HPLC conditions to analyze TMP and SMX; first-order rate constants for abatement, decay, and removal percentages of TMP and SMX in the presence of NOM, phenol and hydroquinone at various pHs; removal and first-order rate constants of TMP in Fe(VI)–phenol system; physicochemical properties of studied natural organic matter; RMSD values obtained in kinetic modeling of TMP and SMX concentration decreases in Fe(VI)–phenol systems (PDF)
