# Supplementary material for: Enhanced Oxidation of Antibiotics by Ferrate Mediated with Natural Organic Matter: Role of Phenolic Moieties
Source: Environ Sci Technol. 2023 Jun 29;57(47):19033–42. doi: 10.1021/acs.est.3c03165 (PMC10862540; doi:10.1021/acs.est.3c03165)
Supplement: Supplementary file 1 — es3c03165_si_001.pdf [file es3c03165_si_001.pdf]

Supporting Information

Enhanced Oxidation of Antibiotics by Ferrate Mediated with Natural Organic Matter: Role of Phenolic Moieties

Binglin Guo[1,2≠], Junyue Wang[3≠], Krishnamoorthy Sathiyan[1], Xingmao Ma[2], Eric Lichtfouse[4], Ching-Hua Huang[3*], and Virender K. Sharma[1*]

[1]Department of Environmental and Occupational Health, School of Public Health, Texas A&M University, College Station, Texas, 77843-8371, USA, vsharma@tamu.edu
[2]Department of Civil and Environmental Engineering, Texas A&M University, College Station, TX, 77843, USA
[3]School of Civil and Environmental Engineering, [4]Georgia Institute of Technology, Atlanta, GA 30332, USA, ching-hua.huang@ce.gatech.edu
[4]Aix-Marseille Univ, CNRS, IRD, INRAE, Coll France, CEREGE, Aix-en-Provence 13100, France

[≠]B.G. and J.W. contributed equally to this paper.

Summary: 28 pages, 14 figures, 13 tables.

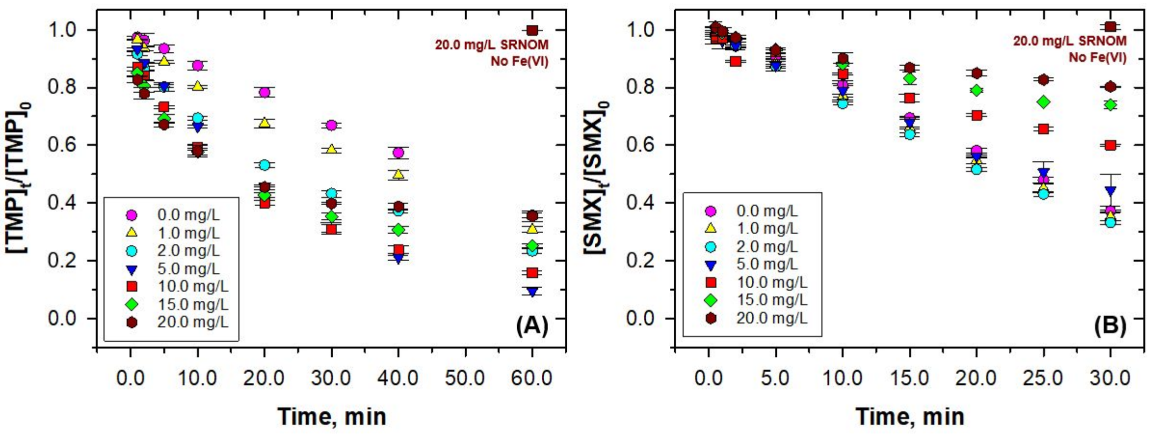

Figure S1. The degradation of representative antibiotics by Fe(VI) in the presence of Suwannee River organic matter (NOM) at **pH 9.0**. **(A)** Trimethoprim (TMP) and **(B)** Sulfamethoxazole (SMX). (Experimental conditions: $[\text{Trimethoprim}]_0$ = $[\text{Sulfamethoxazole}]_0$ = 5.0 μM, $[\text{Fe(VI)}]_0$ = 100.0 μM, pH = 9.0 buffered by 10.0 mM Na_2HPO_4)

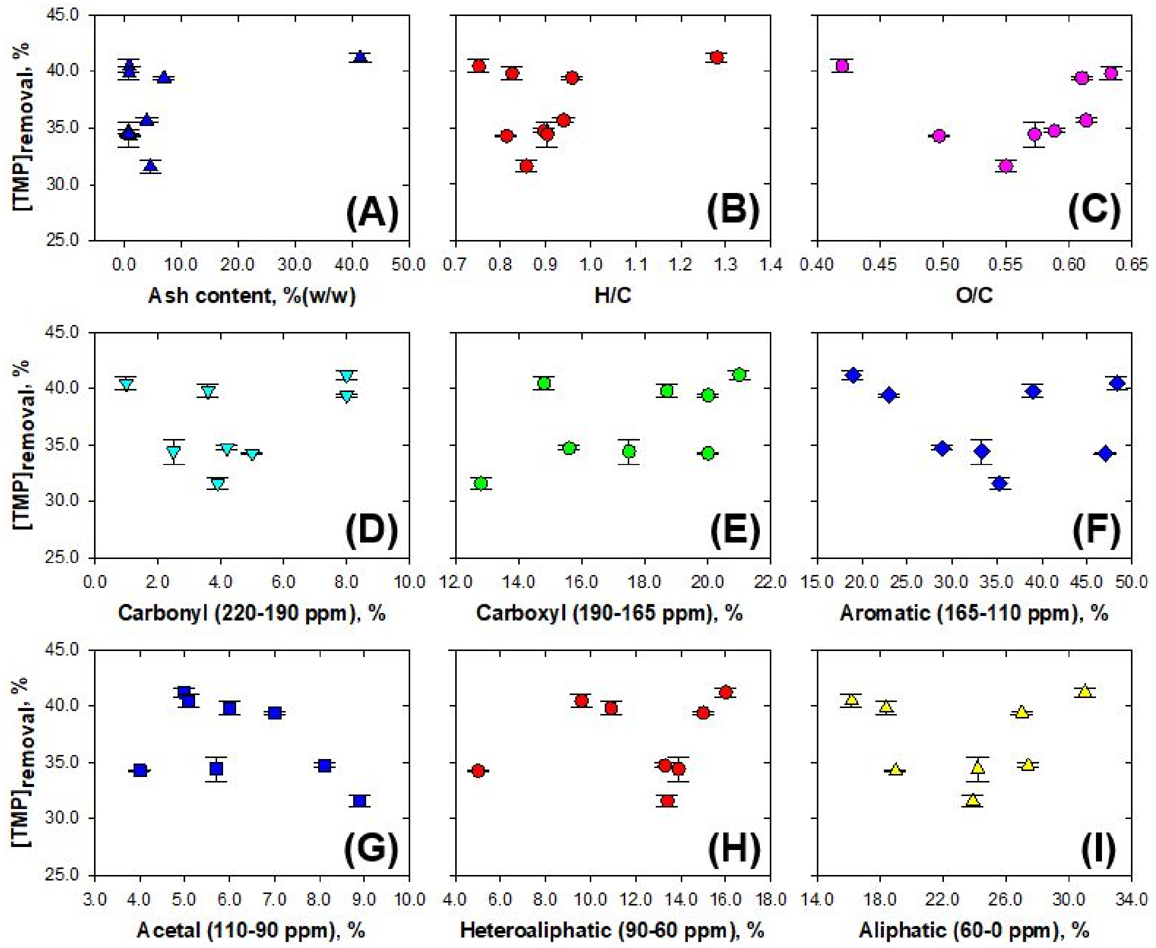

Figure S2. The correlation between the removal of trimethoprim (TMP) at 30 min in the presence of nine standard NOMs and their physiochemical properties. The NOM physiochemical parameters were obtained from IHSS websites (https://humic-substances.org/). In which, data on figures **(A)-(C)** (ash content, H/C and O/C data) were either directly or calculated from elemental analyses; data on figures **(D)-(I)** (percentage of carbon distribution as carbonyl, carboxyl, aromatic, acetal, heteroaliphatic, aliphatic) were acquired by solid-state CPMAS ^{13}C NMR spectra. (Experimental conditions: $[Trimethoprim]_0 = 5.0$ μM, $[Fe(VI)]_0 = 100.0$ μM, pH = 9.0 buffered by 10.0 mM Na_2HPO_4, reaction time = 30.0 min.)

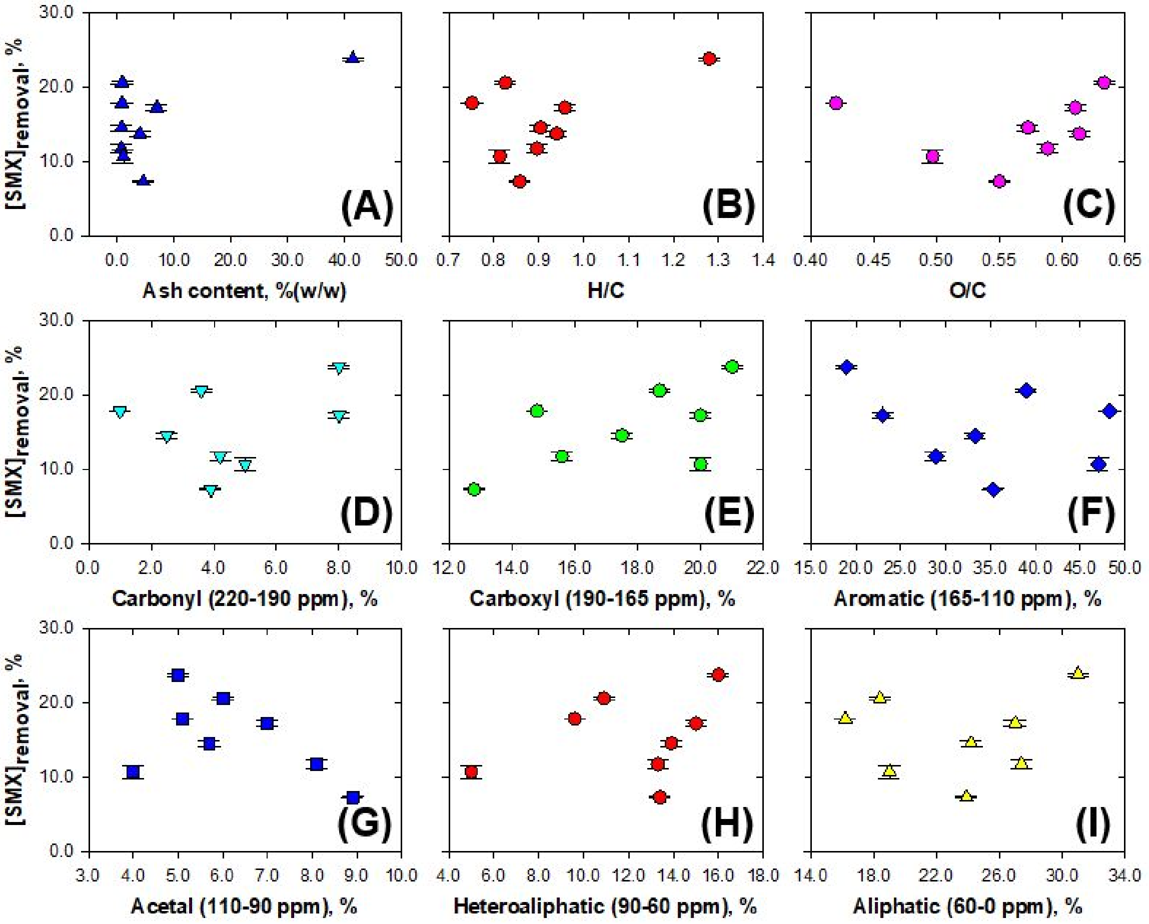

Figure S3. The relationship between the removal of sulfamethoxazole at 15.0 min as affected by nine standard NOMs and their physiochemical properties. The NOM physiochemical parameters were obtained from IHSS websites (https://humic-substances.org/). In which, data on figures **(A)-(C)** (ash content, H/C and O/C data) were either directly or calculated from elemental analyses; data on figures **(D)-(I)** (percentage of carbon distribution as carbonyl, carboxyl, aromatic, acetal, heteroaliphatic, aliphatic) were acquired by solid-state CPMAS ^{13}C NMR spectra. (Experimental conditions: $[\text{Sulfamethoxazole}]_0$ = 5.0 μM, $[\text{Fe(VI)}]_0$ = 100.0 μM, pH = 9.0 buffered by 10.0 mM Na_2HPO_4, reaction time = 15.0 min.)

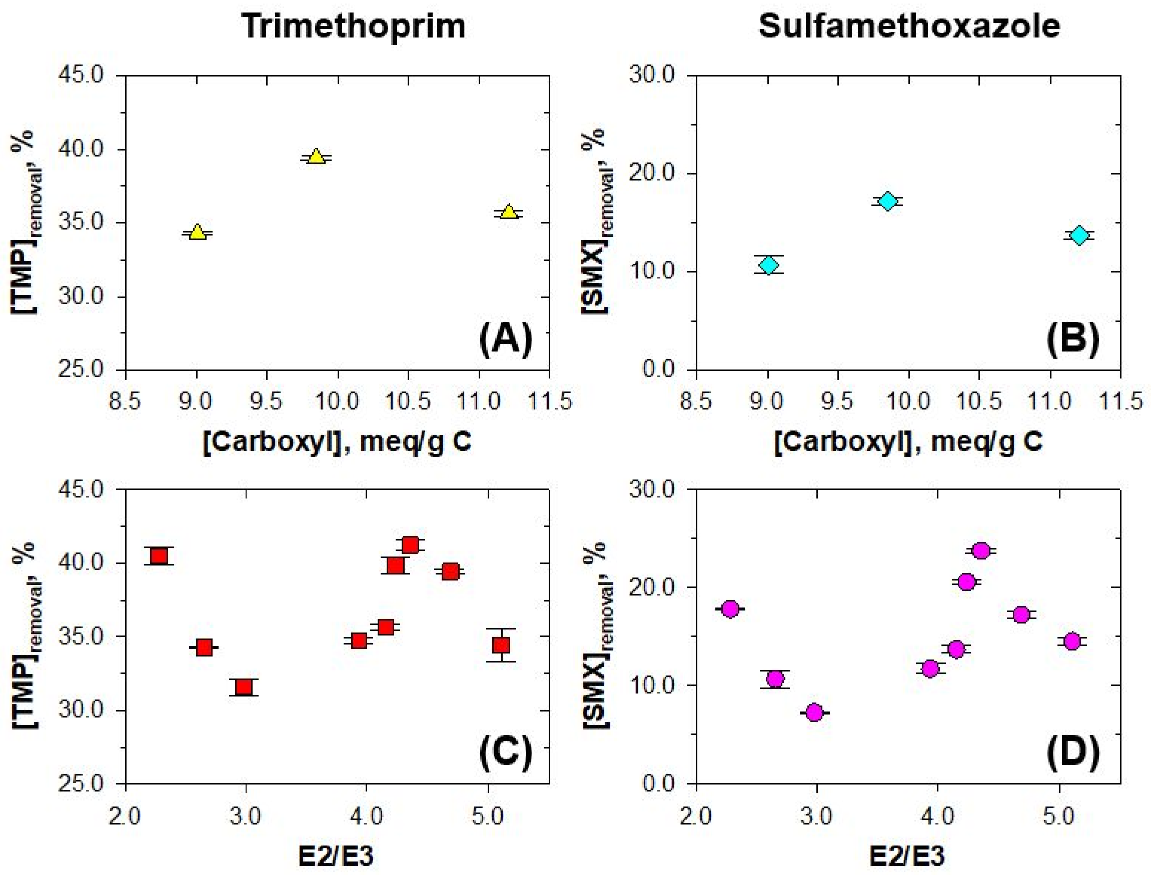

Figure S4. The relationship between the removal of trimethoprim (TMP) at 30.0 min or sulfamethoxazole (SMX) at 15.0 min by Fe(VI) and the physiochemical properties of nine standard NOMs, i.e. between (**A**) removal of trimethoprim and carboxyl content, (**B**) removal of sulfamethoxazole and carboxyl content, (**C**) removal of trimethoprim and E2/E3, and (**D**) removal of sulfamethoxazole and E2/E3, (E2/E3 refers to Abs250/Abs365, given in Table S7, the carboxyl content in meq/g C were obtained by titration method available on IHSS website) (Experimental conditions: $[\text{Trimethoprim}]_0$ = $[\text{Sulfamethoxazole}]_0$ = 5.0 μM, $[\text{Fe(VI)}]_0$ = 100.0 μM, pH = 9.0 buffered by 10.0 mM Na_2HPO_4, reaction time = 30.0 min for trimethoprim and 15.0 min for sulfamethoxazole.)

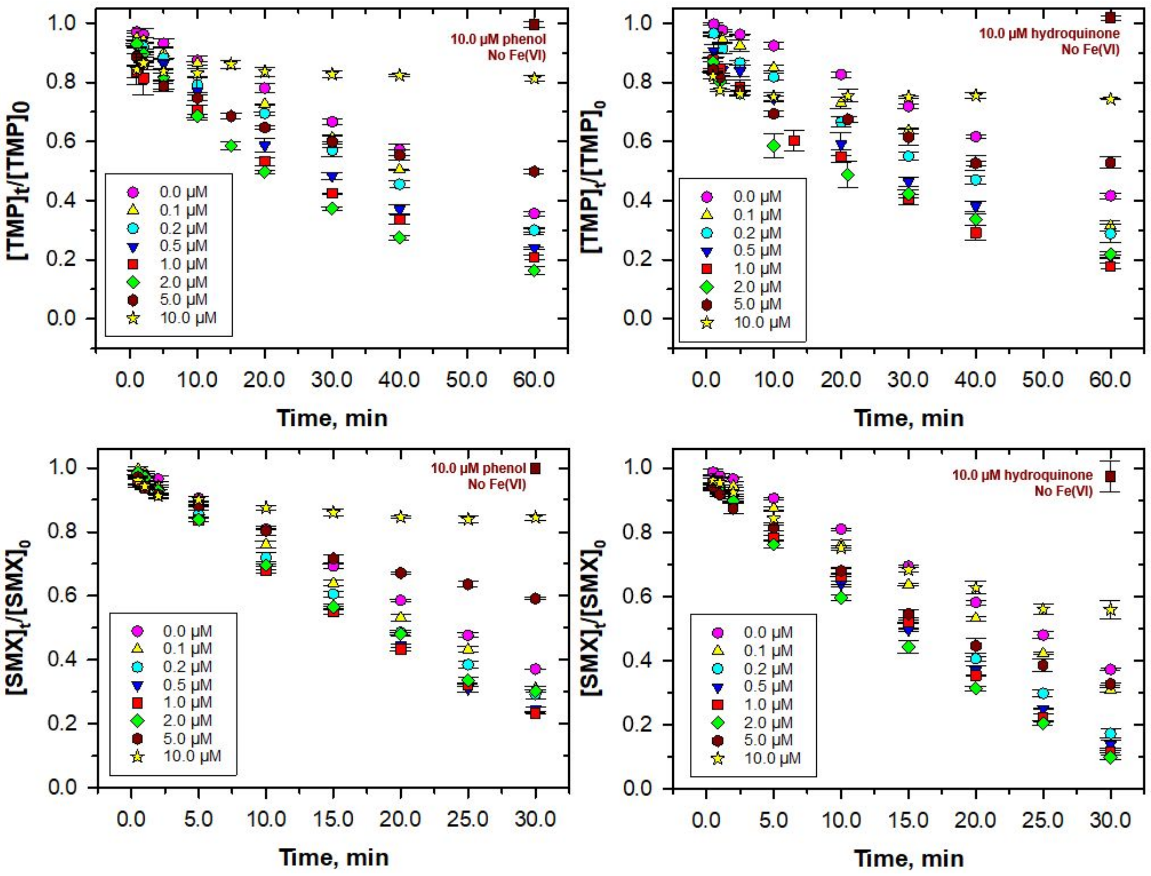

Figure S5. Degradation of antibiotics as a function of time by Fe(VI) in the presence of NOM model compounds at **pH 9.0**. **(A)** Degradation of trimethoprim in the presence of phenol, **(B)** Degradation of trimethoprim in the presence of hydroquinone, **(C)** Degradation of sulfamethoxazole in the presence of phenol, and **(D)** Degradation of sulfamethoxazole in the presence of hydroquinone. (Experimental conditions: $[\text{Trimethoprim}]_0 = [\text{Sulfamethoxazole}]_0 = 5.0$ µM, $[\text{Fe(VI)}]_0 = 100.0$ µM, pH = 9.0 buffered by 10.0 mM Na_2HPO_4.)

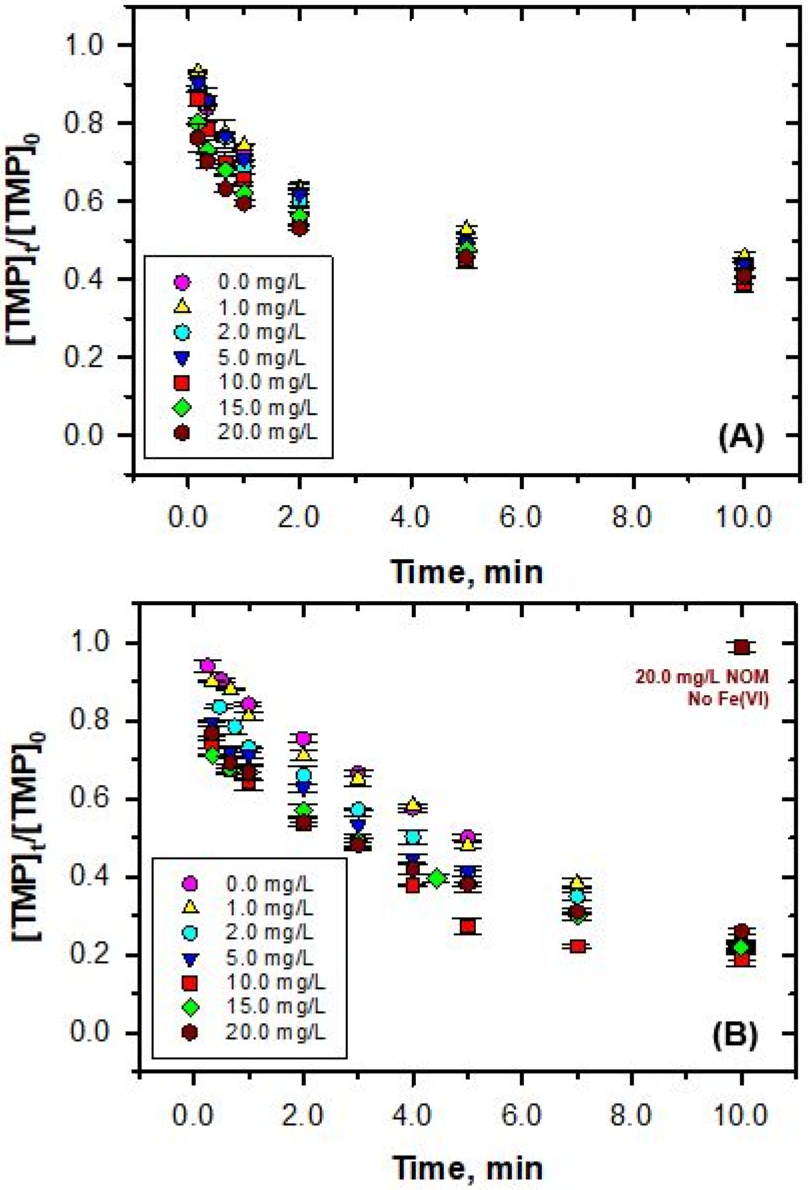

Figure S6. The decrease in concentration of trimethoprim by Fe(VI) in the presence of various concentrations of NOM at different times at (**A**) **pH 7.0** and (**B**) **pH 8.0**. (Experimental conditions: $[Trimethoprim]_0$ = 5.0 μM, $[Fe(VI)]_0$ = 100.0 μM, pH = 8.0 buffered by 10.0 mM Na_2HPO_4.

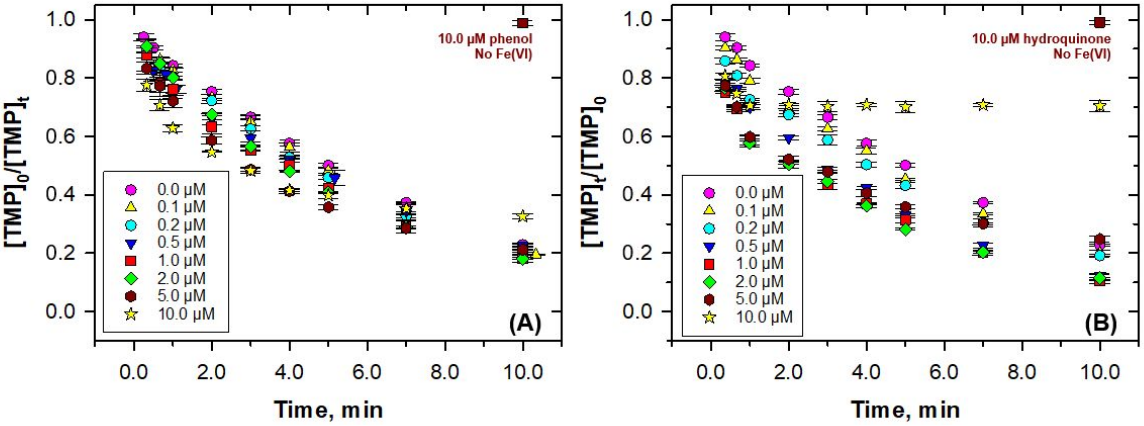

Figure S7. Degradation of trimethoprim (TMP) by Fe(VI) at **pH 8.0** in the presence of model compounds: (A) phenol, (B) hydroquinone. (Experimental conditions: [Trimethoprim]$_0$ = 5.0 µM, [Fe(VI)]$_0$ = 100.0 µM, pH = 8.0 buffered by 10.0 mM Na_2HPO_4)

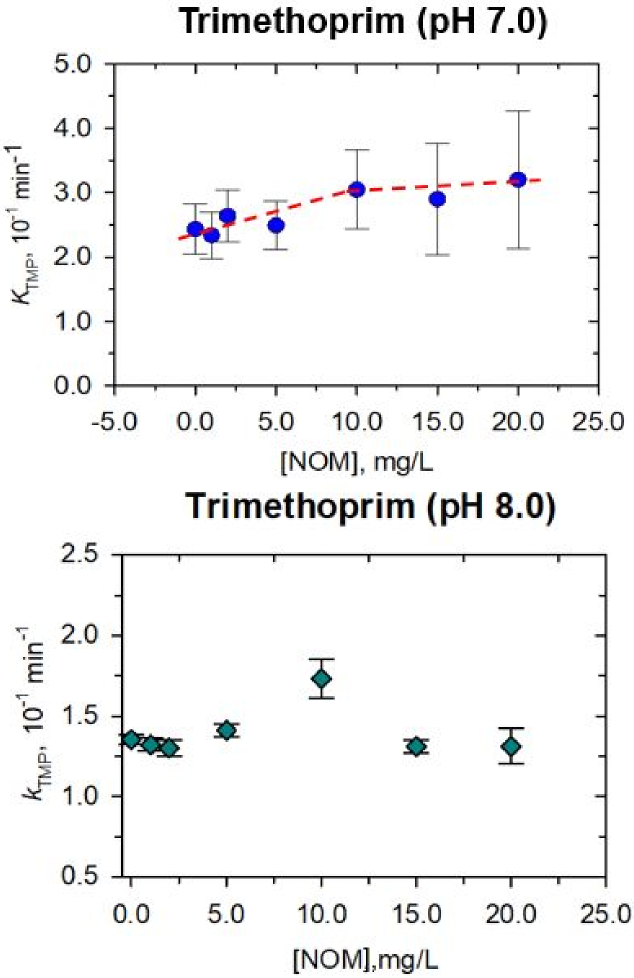

Figure S8. The effects of NOM at different concentrations on the first-order decay rate constants of trimethoprim (TMP) by Fe(VI) at (**A**) pH 7.0, and (**B**) pH 8.0. (Experimental conditions: $[Trimethoprim]_0$ = 5.0 μM, $[SRNOM]_0$ = 1.0-20.0 mg/L, $[Fe(VI)]_0$ = 100.0 μM, buffered by 10.0 mM Na_2HPO_4)

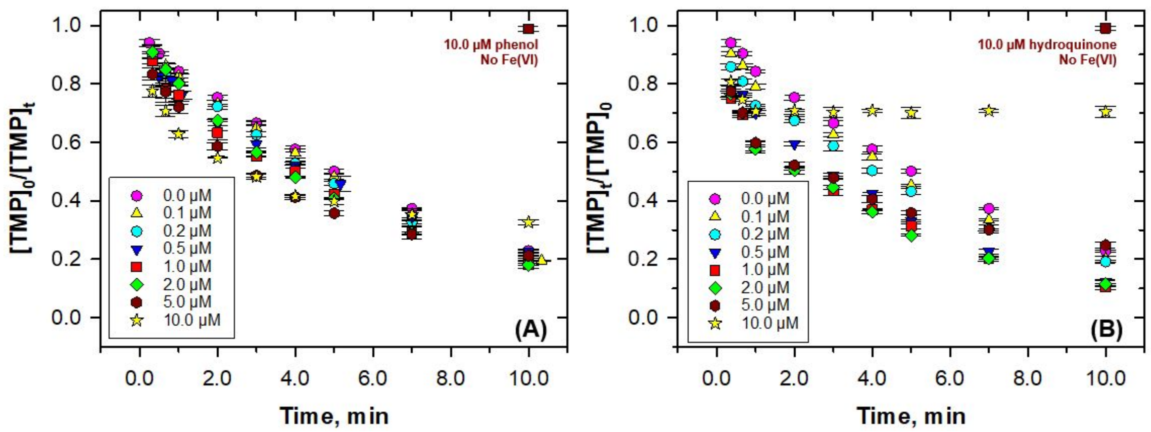

Figure S9. The first order decay of trimethoprim (TMP) decomposed by Fe(VI) in the presence of **(A)** phenol, **(B)** hydroquinone at **pH 8.0**. (Experimental conditions: $[Trimethoprim]_0$ = 5.0 μM, $[Fe(VI)]_0$ = 100.0 μM, pH = 8.0 buffered by 10.0 mM Na_2HPO_4)

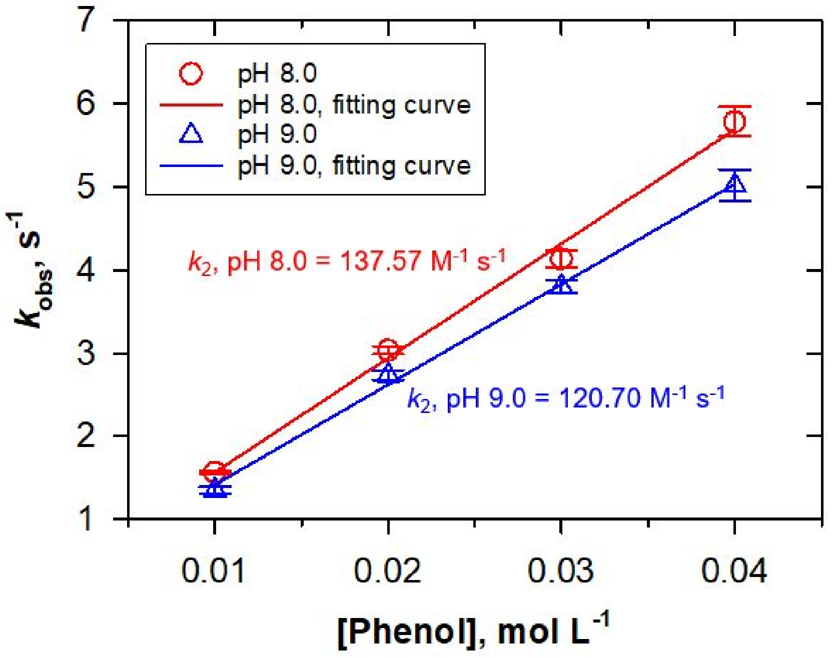

Figure S10. The pseudo first-order rate constant k_{obs}, min^{-1} of the reaction between Fe(VI) and phenol at **pH 8.0** and **pH 9.0**. (R^2 = 0.9974 and 0.9978 for pH 8.0 and pH 9.0 respectively; [Phenol] >> [Fe(VI)], $[Fe(VI)]_0$ = 100.0 μM, pH was maintained by 10.0 mM Na_2HPO_4.)

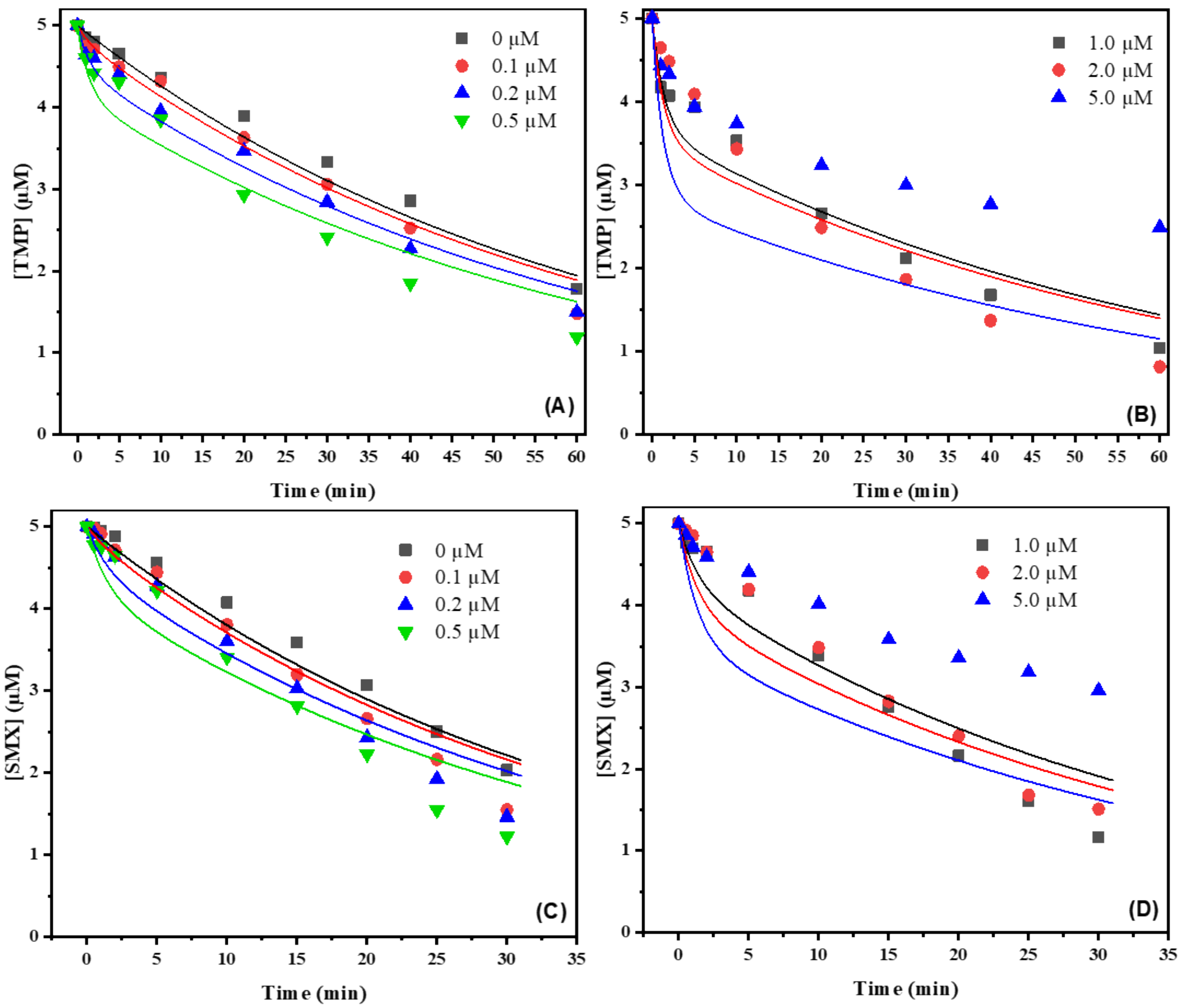

Figure S11. Kinetic modeling for trimethoprim (TMP) **(A)**, **(B)** and sulfamethoxazole (SMX) **(C)**, **(D)** degradation in the absence and presence of phenol (0.1-5.0 μM). (Reaction conditions: $[Trimethoprim]_0 = [Sulfamethoxazole]_0 = 5.0$ μM, $[Fe(VI)]_0 =$ 100.0 μM, pH = 9.0 buffered by 10.0 mM Na_2HPO_4. Symbols represent experimental data and solid lines represent the kinetic modeling.)

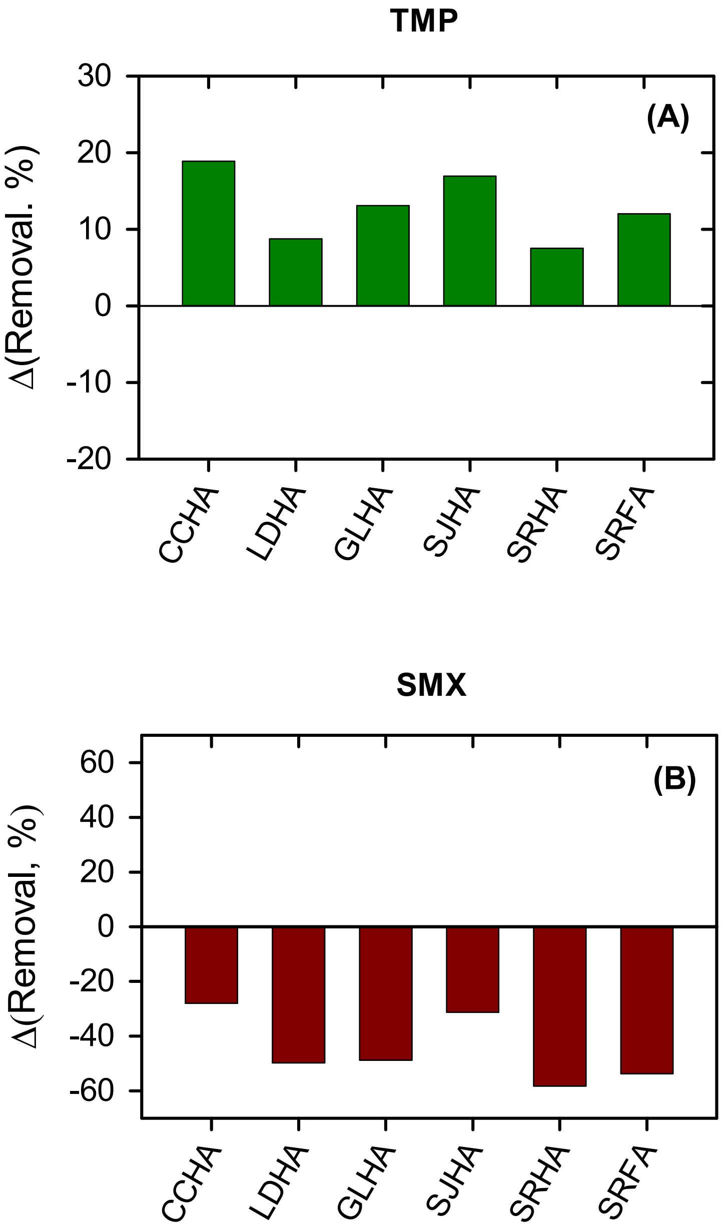

Figure S12. Δ(Removal, %) = Removal(Fe(VI)-trimethoprim/sulfamethoxazole)-Removal(Fe(VI)-trimethoprim/sulfamethoxazole/Organic Matter) for different organic matter of lake water and river at pH 9.0. **(A)** Trimethoprim (TMP) and **(B)** sulfamethoxazole (SMX). CCHA- Crane Creek Humic Acid, LDHA-Lake Dehancy Humic Acid, GLHA-Grass lake Humic Acid; SJHA-St. John River Humic Acid, SRHA-Suwannee River Humic Acid, and SRFA-Suwannee River fulvic Acid). (Experimental conditions: $[\text{Trimethoprim}]_0$ = $[\text{Sulfamethoxazole}]_0$ = 5.0 μM, $[\text{Fe(VI)}]_0$ = 100.0 μM, [NOM] = 10.0 mg/L, Reaction time = 60.0 min, pH = 9.0 buffered by 10.0 mM Na_2HPO_4)

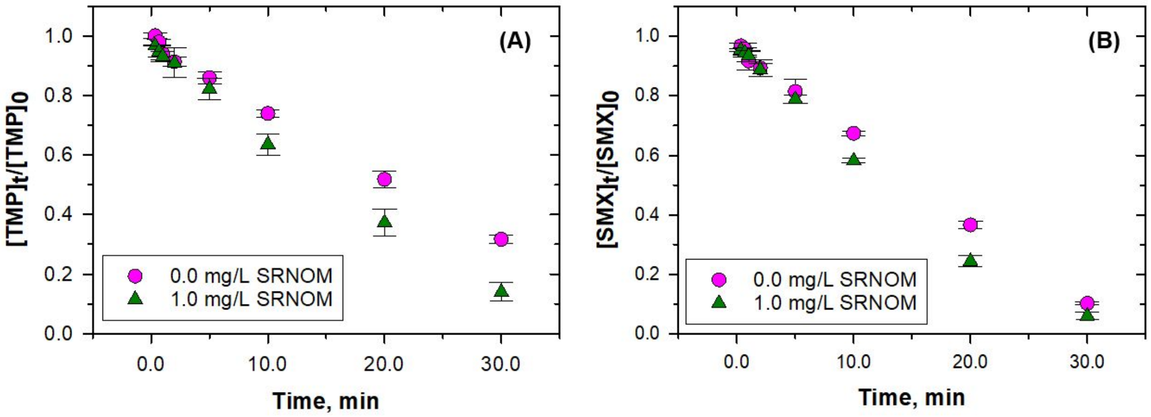

Figure S13. The effects of 1.0 mg/L NOM on the degradation of 1.0 μM micropollutants by Fe(VI), (A) the degradation of trimethoprim (TMP), (B) the degradation of sulfamethoxazole (SMX). (Experimental conditions: $[\text{Trimethoprim}]_0$ = $[\text{Sulfamethoxazole}]_0$ = 1.0 μM, $[\text{SRNOM}]_0$ = 1.0 mg/L, $[\text{Fe(VI)}]_0$ = 100.0 μM, pH = 9.0 buffered by 10.0 mM Na_2HPO_4)

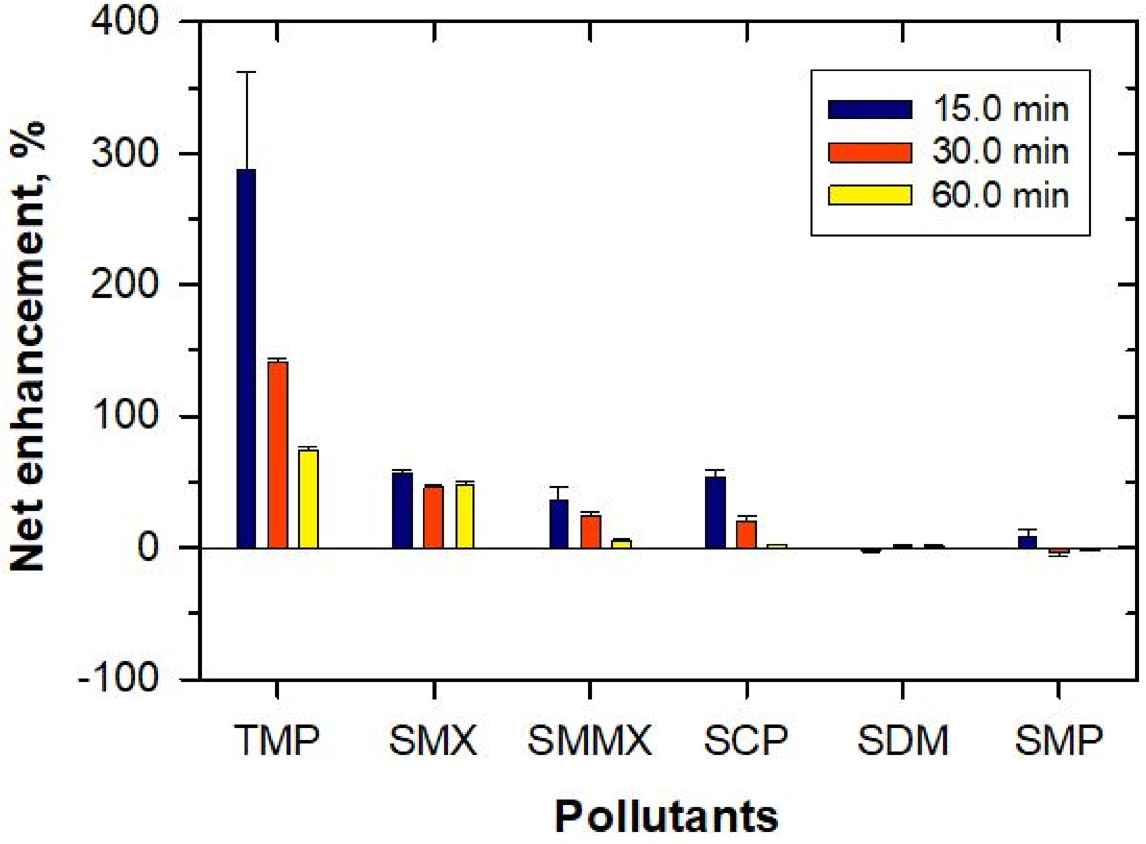

Figure S14. The net enhancement of 1.0 mg/L SRNOM on the removal of multiple micropollutants by Fe(VI). (Experimental conditions: $[\text{Pollutants}]_0$ = 5.0 μM, $[\text{SRNOM}]_0$ = 1.0 mg/L, $[\text{Fe(VI)}]_0$ = 100.0 μM, pH = 9.0 buffered by 10.0 mM Na_2HPO_4).

Table S1. The HPLC (high performance liquid chromatography) analytical conditions for the pollutants in this study

Pollutants	Mobile phase (B) : (A)	Flow rate (mL/min)	Detection Wavelength (nm)	Injection volume (μL)	Retention time (min)
TMP	21:79	1.0	271	20	8.25
SMX	25:75	0.8	271	20	7.76

Table S2. First-order rate constants for the decrease in concentration of trimethoprim (TMP) in the Fe(VI)-trimethoprim-NOM mixed solution at **pH 9.0**. ((Experimental conditions: [Trimethoprim]$_0$ = 5.0 μM, [Fe(VI)]$_0$ = 100.0 μM, **pH = 9.0** buffered by 10.0 mM Na_2HPO_4).

[NOM], mg/L	k_{TMP}, min^{-1}	r^2	[Removal]$_{TMP}$, % (30.0 min)
0.0	(1.48±0.08)×10^{-2}	0.9849	64.3±1.9
1.0	(1.83±0.06)×10^{-2}	0.9956	69.3±1.3
2.0	(2.53±0.16)×10^{-2}	0.9841	76.3±3.3
5.0	(3.83±0.08)×10^{-2}	0.9981	90.6±1.4
10.0	(3.74±0.31)×10^{-2}	0.9795	84.2±4.8
15.0	(3.74±0.51)×10^{-2}	0.9480	74.9±2.2 (20.0 min)
20.0	(3,74±0.83)×10^{-2}	0.8861	74.4±5.0 (20.0 min)

Table S3. First-order rate constants for the decrease in concentrations of trimethoprim (TMP) in the Fe(VI)-trimethoprim-NOM mixed solution at **pH 8.0**. ((Experimental conditions: $[\text{Trimethoprim}]_0 = 5.0$ μM, $[\text{Fe(VI)}]_0 = 100.0$ μM, **pH = 8.0** buffered by 10.0 mM Na_2HPO_4).

[NOM], mg/L	k_{TMP}, min^{-1}	r^2	$[\text{Removal}]_{TMP}$, % (5.0 min)
0.0	$(1.35\pm0.03)\times10^{-1}$	0.99	49.9±0.8
1.0	$(1.32\pm0.04)\times10^{-1}$	0.99	51.9±1.0
2.0	$(1.30\pm0.05)\times10^{-1}$	0.99	59.1±0.6
5.0	$(1.41\pm0.04)\times10^{-1}$	0.99	58.7±1.2
10.0	$(1.73\pm0.12)\times10^{-1}$	0.99	72.8±2.2
15.0	$(1.31\pm0.04)\times10^{-1}$	0.99	62.0±1.9
20.0	$(1.31\pm0.11)\times10^{-1}$	0.97	61.8±0.6

Table S4. First-order rate constants for the decrease in concentration of trimethoprim in the Fe(VI)-trimethoprim-NOM mixed solution at **pH 7.0**. ((Experimental conditions: $[\text{Trimethoprim}]_0$ = 5.0 μM, $[\text{Fe(VI)}]_0$ = 100.0 μM, **pH = 7.0** buffered by 10.0 mM Na_2HPO_4).

[NOM], mg/L	k_{TMP}, min^{-1}	r^2	$[\text{Removal}]_{TMP}$, % (10.0 min)
0.0	$(2.43\pm0.04)\times10^{-1}$	0.92	56.7±0.7
1.0	$(2.34\pm0.04)\times10^{-1}$	0.92	54.2±1.4
2.0	$(2.64\pm0.04)\times10^{-1}$	0.93	57.0±2.5
5.0	$(2.49\pm0.04)\times10^{-1}$	0.93	56.1±1.0
10.0	$(3.04\pm0.06)\times10^{-1}$	0.88	61.2±2.1
15.0	$(2.99\pm0.09)\times10^{-1}$	0.84	59.4±1.8
20.0	$(3.20\pm0.11)\times10^{-1}$	0.80	59.0±0.0

Table S5. First-order rate constants for the decrease in concentration of sulfamethoxazole in the Fe(VI)-sulfamethoxazole-NOM mixed solution at **pH 9.0**. ((Experimental conditions: $[\text{Sulfamethoxazole}]_0$ = 5.0 μM, $[\text{Fe(VI)}]_0$ = 100.0 μM, **pH = 9.0** buffered by 10.0 mM Na_2HPO_4).

[NOM], mg/L	k_{SMX}, min^{-1}	r^2	$[\text{Removal}]_{SMX}$ % (30.0 min)
0.0	$(2.92±0.16)×10^{-2}$	0.9831	63.8±0.7
1.0	$(3.14±0.13)×10^{-2}$	0.9910	64.8±103
2.0	$(3.35±0.11)×10^{-2}$	0.9937	66.8±1.7
5.0	$(2.68±0.07)×10^{-2}$	0.9961	55.4±1.2
10.0	$(1.61±0.11)×10^{-2}$	0.9760	39.9±0.6
15.0	$(1.10±0.05)×10^{-2}$	0.9850	26.0±0.6
20.0	$(0.76±0.06)×10^{-2}$	0.9606	19.7±0.2

Table S6. The removal percentage of trimethoprim (TMP) at 30 min and sulfamethoxazole (SMX) at 15 min by Fe(VI) in the presence of 9 standard NOMs. (Experimental conditions: $[\text{Trimethoprim}]_0$ = $[\text{Sulfamethoxazole}]_0$ = 5.0 μM, $[\text{Fe(VI)}]_0$ = 100.0 μM, pH = 9.0 buffered by 10.0 mM Na_2HPO_4, reaction time = 60.0 min.)

IHSS Standards	Types	$[\text{TMP}]_{\text{Removal}}$, %	$[\text{SMX}]_{\text{Removal}}$, %
1R108N	Nordic Lake I NOM	41.23 ± 0.36	23.8 ± 0.20
2R101N	Suwannee River II NOM	35.65 ± 0.19	13.72 ± 0.38
3S101F	Suwannee River III FA	34.72 ± 0.19	11.73 ± 0.54
3S101H	Suwannee River III HA	31.59 ± 0.55	7.28 ± 0.07
1R101N	Suwannee River I NOM	39.42 ± 0.17	17.23 ± 0.41
5S102H	Elliott Soil V HA	40.49 ± 0.60	17.83 ± 0.05
2S103F	Pahokee Peat II FA	39.82 ± 0.57	20.56 ± 0.25
1S103H	Pahokee Peat I HA	34.28 ± 0.09	10.69 ± 0.92
5S102F	Elliott Soil V FA	34.43 ± 1.10	14.54 ± 0.36

Table S7. Molecular compositions of nine IHSS standard NOMs used in the study.

IHSS standards	Name	Ash % (w/w)	H/C	O/C	Carboxyl meq/g C	Phenolic meq/g C	Carbonyl 220-190 ppm	Carboxyl 190-165 ppm	Aromatic 165-110 ppm	Acetal 110-90 ppm	Heteroaliphatic 90-60 ppm	Aliphatic 60-0 ppm	E2/E3
1R108N	Nordic Lake I NOM	41.40	1.28	N/A	nd	5.84	8.00	21.00	19.00	5.00	16.00	31.00	4.35
2R101N	Suwannee River II NOM	4.01	0.94	0.61	11.21	2.47	N/A	N/A	N/A	N/A	N/A	N/A	4.15
3S101F	Suwannee River III FA	0.78	0.90	0.59	N/A	2.98	4.20	15.60	28.90	8.10	13.30	27.40	3.94
3S101H	Suwannee River III HA	4.62	0.86	0.55	N/A	3.12	3.90	12.80	35.30	8.90	13.40	23.90	2.98
1R101N	Suwannee River I NOM	7.00	0.96	0.61	9.85	3.97	8.00	20.00	23.00	7.00	15.00	27.00	4.69
5S102H	Elliott Soil V HA	0.88	0.75	0.42	N/A	5.01	1.00	14.80	48.30	5.10	9.60	16.20	2.28
2S103F	Pahokee Peat II FA	0.90	0.83	0.63	nd	5.29	3.60	18.70	39.00	6.00	10.90	18.40	4.24
1S103H	Pahokee Peat I HA	1.12	0.81	0.50	9.01	1.91	5.00	20.00	47.00	4.00	5.00	19.00	2.65
5S102F	Elliott Soil V FA	0.80	0.90	0.57	N/A	2.86	2.50	17.50	33.30	5.70	13.90	24.20	5.11

E2/E3 values (Abs250/Abs365) were calculated from the full wavelength scans of 9 standard NOM solutions (10.0 mg/L). Other data were obtained from https://humic-substances.org/

"N/A" means that the data is not available; "nd" means that an item was not determined. The relative abundance of functional groups shown in Column 8-13 were obtained by ^{13}C NMR.

Table S8. First-order rate constants for the decrease in concentration of trimethoprim (TMP) in the Fe(VI)-trimethoprim-Phenol mixed solution at **pH 9.0**. ((Experimental conditions: [Trimethoprim]$_0$ = 5.0 μM, [Fe(VI)]$_0$ = 100.0 μM, **pH = 9.0** buffered by 10.0 mM Na_2HPO_4).

[Phenol], μM	k_{TMP}, min^{-1}	r^2	[Removal]$_{TMP}$, % (30.0 min)
0.0	$(1.48\pm0.08)\times10^{-2}$	0.9849	64.3±1.9
0.1	$(1.74\pm0.50)\times10^{-2}$	0.9861	70.1±3.0
0.2	$(1.86\pm0.20)\times10^{-2}$	0.9750	70.0±1.3
0.5	$(2.35\pm0.09)\times10^{-2}$	0.9900	76.1±1.4
1.0	$(2.53\pm0.20)\times10^{-2}$	0.9755	79.1±2.5
2.0	$(3.22\pm0.09)\times10^{-2}$	0.9980	84.6±1.0
5.0	$(1.21\pm0.17)\times10^{-2}$	0.8880	50.2±0.8
10.0	Data do not fit		

Table S9. First-order rate constants for the decrease in concentration of trimethoprim (TMP) in the Fe(VI)-trimethoprim-Hydroquinone mixed solution at **pH 9.0**. (Experimental conditions: $[\text{Trimethoprim}]_0$ = 5.0 μM, $[\text{Fe(VI)}]_0$ = 100.0 μM, **pH = 9.0** buffered by 10.0 mM Na_2HPO_4).

[Hydroquinone], μM	k_{TMP}, min^{-1}	r^2	$[\text{Removal}]_{TMP}$, % (30.0 min)
0.0	$(1.30\pm0.08)\times10^{-2}$	0.9860	63.3±2.1
0.1	$(1.67\pm0.08)\times10^{-2}$	0.9890	68.4±4.3
0.2	$(1.93\pm0.06)\times10^{-2}$	0.9951	71.2±2.1
0.5	$(2.39\pm0.13)\times10^{-2}$	0.9893	70.1±1.3
1.0	$(2.85\pm0.18)\times10^{-2}$	0.9840	82.3±4.6
2.0	$(1.94\pm0.06)\times10^{-2}$	0.9951	79.3±3.6
5.0	$(1.01\pm0.26)\times10^{-2}$	0.8100	82.2±3.6
10.0	Data do not fit		

Table S10. First-order rate constants for the decrease in concentration of sulfamethoxazole in the Fe(VI)-sulfamethoxazole-Phenol mixed solution at **pH 9.0**. ((Experimental conditions: $[Sulfamethoxazole]_0$ = 5.0 μM, $[Fe(VI)]_0$ = 100.0 μM, **pH = 9.0** buffered by 10.0 mM Na_2HPO_4).

[Phenol], μM	k_{SMX}, min^{-1}	r^2	$[Removal]_{SMX}$, % (30.0 min)
0.0	$(2.90\pm0.16)\times10^{-2}$	0.9829	63.0±1.2
0.1	$(3.35\pm0.15)\times10^{-2}$	0.9881	69.3±1.7
0.2	$(2.71\pm0.13)\times10^{-2}$	0.9930	70.1±1.9
0.5	$(4.24\pm0.18)\times10^{-2}$	0.9991	75.5±2.1
1.0	$(4.29\pm0.17)\times10^{-2}$	0.9920	77.7±1.0
2.0	$(3.95\pm0.10)\times10^{-2}$	0.9950	69.8±0.9
5.0	$(1.75\pm0.10)\times10^{-2}$	0.9850	31.9±1.9
10.0	Data do not fit		15.5±.80

Table S11. First-order rate constants for the decrease in concentration of sulfamethoxazole in the Fe(VI)-sulfamethoxazole-Hydroquinone mixed solution at **pH 9.0**. ((Experimental conditions: $[Sulfamethoxazole]_0$ = 5.0 μM, $[Fe(VI)]_0$ = 100.0 μM, **pH = 9.0** buffered by 10.0 mM Na_2HPO_4).

[Hydroquinone], μM	k_{SMX}, min^{-1}	r^2	$[Removal]_{SMX}$, % (30.0 min)
0.0	$(2.90\pm0.16)\times10^{-2}$	0.9829	63.0±1.2
0.1	$(3.35\pm0.17)\times10^{-2}$	0.9850	61.0±2.3
0.2	$(4.67\pm0.25)\times10^{-2}$	0.9871	82.8±3.6
0.5	$(5.14\pm0.28)\times10^{-2}$	0.9870	86.2±2.6
1.0	$(5.24\pm0.39)\times10^{-2}$	0.9785	89.3±5.9
2.0	$(5.92\pm0.30)\times10^{-2}$	0.9900	90.2±5.6
5.0	$(3.79\pm0.11)\times10^{-2}$	0.9950	67.3±0.8
10.0	$(2.12\pm0.12)\times10^{-2}$	0.9792	44.1±2.1

Table S12. First-order rate constants for the decrease in concentration of trimethoprim (TMP) in the Fe(VI)-trimethoprim-Phenol mixed solution at **pH 8.0**. ((Experimental conditions: $[Trimethoprim]_0$ = 5.0 μM, $[Fe(VI)]_0$ = 100.0 μM, **pH = 8.0** buffered by 10.0 mM Na_2HPO_4).

[Phenol], μM	k_{TMP}, min^{-1}	r^2	$[Removal]_{TMP}$, % (10.0 min)
0.0	$(1.37\pm0.04)\times10^{-1}$	0.9972	77.1±1.0
0.1	$(1.42\pm0.05)\times10^{-1}$	0.9920	81.5±1.4
0.2	$(1.51\pm0.05)\times10^{-1}$	0.9930	81.1±0.6
0.5	$(1.44\pm0.08)\times10^{-1}$	0.9815	77.2±2.0
1.0	$(1.67\pm0.10)\times10^{-1}$	0.9835	81.8±5.9
2.0	$(1.67\pm0.10)\times10^{-1}$	0.9830	81.2±5.6
5.0	$(1.85\pm0.16)\times10^{-1}$	0.8870	78.8±6.0
10.0	$(1.41\pm0.26)\times10^{-1}$	0.8300	67.3±5.2

Table S13. Root-mean-square deviation (RMSD) values for kinetic modeling of trimethoprim (TMP) and sulfamethoxazole (SMX) degradation in the Fe(VI)-phenol system. (Experimental conditions: $[\text{Trimethoprim}]_0 = [\text{Sulfamethoxazole}]_0 = 5.0$ μM, $[\text{Fe(VI)}]_0 = 100.0$ μM, **pH = 9.0** buffered by 10.0 mM Na_2HPO_4).

[phenol], μM	RMSD for TMP	RMSD for SMX
0.0	$(1.35 \pm 0.08) \times 10^{-1}$	0.045
0.1	0.047	0.087
0.2	0.046	0.106
0.5	0.098	0.123
1.0	0.130	0.158
2.0	0.125	0.129
